# The Evolution of Ultraconserved Elements in Vertebrates

**DOI:** 10.1093/molbev/msae146

**Published:** 2024-07-16

**Authors:** Mitchell Cummins, Cadel Watson, Richard J Edwards, John S Mattick

**Affiliations:** School of Biotechnology and Biomolecular Sciences, UNSW Sydney, Sydney, NSW 2052, Australia; School of Engineering, UNSW Sydney, Sydney, NSW 2052, Australia; School of Biotechnology and Biomolecular Sciences, UNSW Sydney, Sydney, NSW 2052, Australia; School of Biotechnology and Biomolecular Sciences, UNSW Sydney, Sydney, NSW 2052, Australia

**Keywords:** ultraconserved elements, tetrapods, conservation, noncoding

## Abstract

Ultraconserved elements were discovered two decades ago, arbitrarily defined as sequences that are identical over a length ≥ 200 bp in the human, mouse, and rat genomes. The definition was subsequently extended to sequences ≥ 100 bp identical in at least three of five mammalian genomes (including dog and cow), and shown to have undergone rapid expansion from ancestors in fish and strong negative selection in birds and mammals. Since then, many more genomes have become available, allowing better definition and more thorough examination of ultraconserved element distribution and evolutionary history. We developed a fast and flexible analytical pipeline for identifying ultraconserved elements in multiple genomes, *dedUCE*, which allows manipulation of minimum length, sequence identity, and number of species with a detectable ultraconserved element according to specified parameters. We suggest an updated definition of ultraconserved elements as sequences ≥ 100 bp and ≥97% sequence identity in ≥50% of placental mammal orders (12,813 ultraconserved elements). By mapping ultraconserved elements to ∼200 species, we find that placental ultraconserved elements appeared early in vertebrate evolution, well before land colonization, suggesting that the evolutionary pressures driving ultraconserved element selection were present in aquatic environments in the Cambrian–Devonian periods. Most (>90%) ultraconserved elements likely appeared after the divergence of gnathostomes from jawless predecessors, were largely established in sequence identity by early Sarcopterygii evolution—before the divergence of lobe-finned fishes from tetrapods—and became near fixed in the amniotes. Ultraconserved elements are mainly located in the introns of protein-coding and noncoding genes involved in neurological and skeletomuscular development, enriched in regulatory elements, and dynamically expressed throughout embryonic development.

## Introduction

Ultraconserved elements (UCEs) were discovered independently by the Haussler and Mattick groups, who reported their existence jointly in 2004 (Bejerano et al. 2004). UCEs were defined at the time as DNA segments with 100% identity among human, rat, and mouse genomes, identifying 481 sequences ≥ 200 bp and over 5,000 ≥ 100 bp, nearly all of which are nonprotein-coding and also highly conserved in chicken (Bejerano et al. 2004).

A subsequent analysis, following the availability of more genome sequences, revealed over 2,000 sequences ≥ 200 bp and almost 14,000 sequences ≥ 100 bp that are identical in at least three of five placental mammals (human, mouse, rat, dog, and cow) (Stephen et al. 2008). This study also showed that there was a massive genome-wide acquisition, rapid evolution relative to protein-coding sequences, and size expansion of UCEs during tetrapod evolution, likely under positive selection, followed by an abrupt slowdown of the molecular clock in the amniotes (Stephen et al. 2008). That is, these sequences evolved rapidly during vertebrate evolution and then became almost frozen in reptiles, birds, and mammals, the latter exhibiting ∼0.01 substitutions per site per 100 million years, an order of magnitude slower than protein-coding sequences (∼0.1 substitutions per site per 100 million years) (Stephen et al. 2008).

A broader study involving a 28-way alignment of vertebrate genomes came to a similar conclusion, namely that almost all UCEs are conserved in amniotes, before which their alignability decays more rapidly than coding exons (Miller et al. 2007). This evolutionary history is indicative of genome-wide functional exaptation followed by strong purifying selection (Stephen et al. 2008), a conclusion supported by analyses of the derived allele frequency spectrum (Katzman et al. 2007). Following publication of the elephant shark genome, it was discovered that ∼40% of the ∼14,000 UCEs could be mapped at ∼80% sequence identity (Wang et al. 2008). This was a greater number mapped and higher sequence identity than pufferfish, a closer relative to human, suggesting earlier evolutionary appearance than first thought (Wang et al. 2008). It was later discovered that ancient conserved noncoding elements have evolved rapidly in the teleost fishes (Lee et al. 2011).

Other criteria have been used to define highly conserved nongenic vertebrate sequences that are similar to UCEs but do not require 100% identity, variously as sequences ≥ 100 bp and ≥70% identity between human and mouse (CNGs) (Dermitzakis et al. 2002, 2005), sequences ≥ 40 bp with ≥65% identity in at least four genomes (Woolfe et al. 2005, 2007), and ultraconserved regions (UCRs) with ≥95% identity over 50 bp between human and mouse genomes that overlap with sequences conserved between human and pufferfish (Sandelin et al. 2004).

UCEs are almost entirely located in nonprotein-coding regions (Bejerano et al. 2004), with ∼77% within intergenic or intronic sequences, 20% overlapping exon-intron boundaries, and only 3% located entirely within a protein-coding exon (Stephen et al. 2008; Snetkova et al. 2022). They are depleted among segmental duplications and copy number variants (Derti et al. 2006). UCEs have been found to predominantly lie within topologically associated domains (McCole et al. 2018), suggesting a function in genome organization, consistent with enhancer activity. UCEs are often associated with enhancers—although enhancer function does not require perfect sequence conservation (Snetkova et al. 2021)—with many clustered around important developmental and neurological genes (Sandelin et al. 2004; Snetkova et al. 2022). Some UCEs are derived from retrotransposons (Bejerano et al. 2006), but the majority have different sequences despite showing similar evolutionary history, which suggests sequence constraints that seem, paradoxically, to be primary sequence agnostic.

The initial definition of UCEs was, by necessity at the time, arbitrary. However, many more vertebrate genomes have since become available, which affords the opportunity to examine their phylogenetic distribution and evolutionary history in more detail. Furthermore, a greater amount of genomic and molecular data are now available, allowing for extensive comparisons between UCEs and other genomic features.

Herein, we introduce a new computational tool for UCE identification, called *dedUCE*, and redefine UCEs in a set of placental mammals. We examine the evolutionary history of these UCEs throughout vertebrate evolution (and beyond), and explore their characteristics and human genome correlates. Furthermore, we intersect UCE coordinates with multiple regulatory and RNA-sequencing datasets, and find that UCEs are associated with neurological and skeletomuscular development, cis-regulatory elements, and are dynamically expressed throughout embryonic development.

## Results

### A New Computational Tool for UCE Identification

A number of computational approaches have been used to identify UCEs. A popular approach is based on sliding windows of conservation. These can be broadly categorized into alignment-based methods and suffix array/k-mer methods. The former utilizes sliding windows or read-mapping of the desired size to identify sequences that meet the conservation criteria, but require UCEs to be detected in specific genomes and risk being disrupted by genetic rearrangements and/or assembly errors (Stephen et al. 2008; Faircloth et al. 2012). The latter are more flexible, but previous implementations have required pairwise comparisons between all genomes in the test set (Christley et al. 2008; Reneker et al. 2012), and no existing method is efficient for analyzing large numbers of genomes in parallel.

**Fig. 1.**
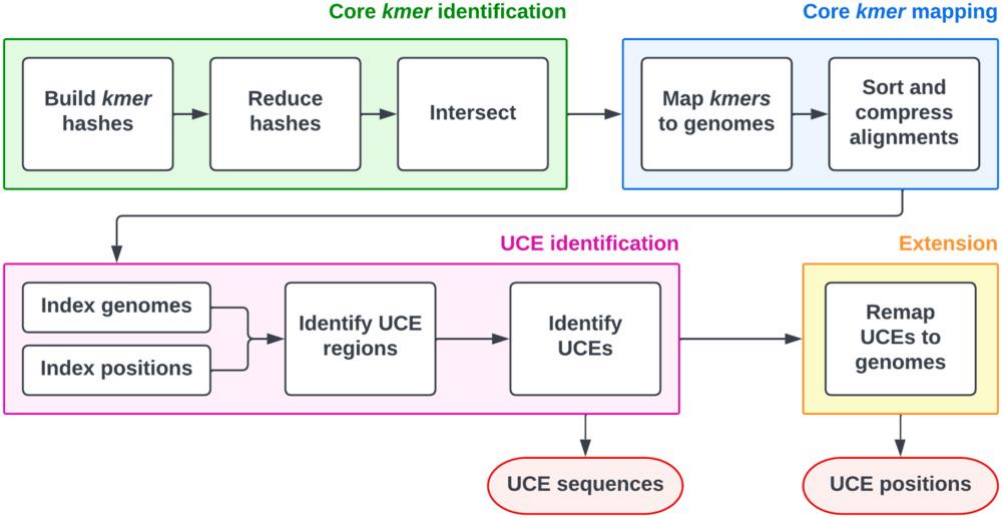
Summary of the *dedUCE* algorithm. The algorithm can be grouped into four stages: (i) identification of core k-mers: using fast k-mer hashing, find all k-mers of a certain length present in X/Y input genomes; (ii) mapping of all positions in which these core k-mers appear in the input genomes within a specified sequence identity threshold; (iii) merging of overlapping core k-mers and identification of all UCEs that meet the specified parameters; and (iv) extension: find all appearances of the UCEs in the input genomes with a lower sequence identity threshold.

We have developed a new program for identifying UCEs, *dedUCE* (available at https://github.com/slimsuite/deduce). *dedUCE* is a fast and flexible k-mer-based method for identifying UCEs in multiple genomes. The *dedUCE* program allows for manipulation of the key variables: minimum UCE length, minimum sequence identity, and minimum number of species satisfying the two parameters. The workflow of *dedUCE* is shown in Fig. 1. Details of the program, validation datasets, and comparisons to other programs are in supplementary File S1, Supplementary Material online. To validate *dedUCE*, we sought to replicate lists of UCE from published studies (supplementary table S1, Supplementary Material online) and could identify, on average, 99% of published UCEs investigated, and found that *dedUCE* outperformed other tools (supplementary table S2, Supplementary Material online), whilst significantly improving speed.

### A Formal Definition of UCEs in Placental Mammals

To define placental mammal UCEs using *dedUCE*, we used the human genome as reference and selected one representative species from each of the 19 mammalian placental orders, making a total of 20 genomes in our definition set (supplementary table S3, Supplementary Material online). Where multiple species were available within an order, the selection of the representative species was based on the best genome coverage and sequence quality. Initially, we used a minimum length of 100 bp and a minimum occurrence in at least 50% of species.

We chose 100 bp as a minimum length for two reasons. First, based on a previous definition of UCEs in placental mammals, elements < 100 bp were found to be a mix of UCEs and elements evolving at a faster rate (Stephen et al. 2008). Second, a 100 bp minimum allows for ease of calculations across different sequence identity and species settings. We chose a minimum of 50% species to provide the necessary flexibility to allow for lineage-specific loss of UCEs, differing rates of evolution or number of generations among species, and detection failures due to errors in lower quality of genome assemblies, whilst still occurring in the majority of species.

**Fig. 2.**
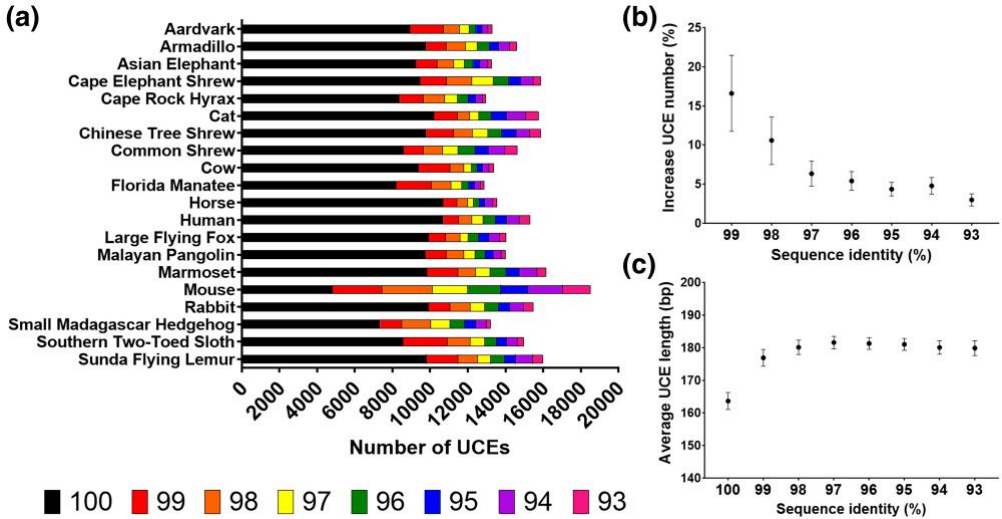
Ultraconserved elements (UCEs) in placental mammals. UCEs were identified using *dedUCE* in 20 species spread across placental mammalian orders. a) The majority of UCEs are found as exact matches in most placental mammal species in the set. Colors indicate sequence identity to UCE sequence. b) The largest increase in UCE number is found when allowing for a single mismatch between the UCE sequence and the sequence in any species. c) The average length of UCEs peaks at 97% sequence identity. Panels b) and c) show mean ± 95% CI.

We found that most UCEs (∼9,200 per species) could be identified in at least 50% of species at 100% sequence identity (Fig. 2a), with the largest increase in UCE number (∼1,400 per species) found when the minimum sequence identity is reduced to 99% (Fig. 2b). Subsequent decreases down to 97% sequence identity yield smaller increases, following which there is a baseline of ∼3% to 5% increase in UCE number for every 1% decrease in minimum sequence identity.

The number of UCE bases (supplementary fig. S1, Supplementary Material online) also follows this distribution. The average UCE length (Fig. 2c) reached a peak in size of ∼180 bp at 97% minimum sequence identity, then remained stable. The majority of UCEs by this definition are between 100 and 200 bp in length (supplementary fig. S2, Supplementary Material online). Graphs for the number of UCEs identified, number of UCE bases, and average UCE length with individual species labeled are provided in supplementary fig. S3, Supplementary Material online.

We next examined the optimal number of species for defining UCEs in placental mammals. We used three incidence criteria (≥50%, ≥ 80%, or 100% of species) of UCEs all with a minimum sequence identity of 97% and minimum length of 100 bp. We found 12,813 UCEs in placental mammals when requiring >97% sequence identity in 50% of species, 3,397 in 80% of species, and 279 in 100% of species.

We then mapped the human UCE sequences against all species in the placental definition set and could identify (minimum mapping requirement ≥ 30 bp, *e*-value < 0.01), on average, 98.6% of the 50% species UCEs at an average sequence identity of 98.4% in each species (supplementary fig. S4a, b, Supplementary Material online). Most UCEs could be mapped in at least 19 of the 20 species in the set, mostly at 98% to 100% sequence identity (supplementary fig. S4c to e, Supplementary Material online). Similar high mapping rates were also found in the 80% set (98.9% of UCEs at 99.5% sequence identity) and the 100% set (99.3% UCEs at 99.8% sequence identity) (supplementary fig. S4g, h, Supplementary Material online).

We also mapped the three sets of UCEs against a set of other placental species (supplementary table S4, Supplementary Material online) and found that similar numbers of UCEs could be mapped, with similar sequence identity, confirming the generalizability of the sets (supplementary fig. S4a to h, Supplementary Material online). We confirmed that UCE core regions were more highly conserved than UCE flanks (supplementary fig. S5a to c, Supplementary Material online) as has been previously reported for UCE sets (Stephen et al. 2008).

On this basis, we settled on a definition of the standard set of mammalian UCEs as those of length ≥ 100 bp with ≥97% sequence identity in at least 50% of mammalian placental orders. The list of the standard set of UCEs with human genome coordinates, grouped by chromosome, is provided in supplementary File S2, Supplementary Material online. The 80% and 100% sets are provided in supplementary Files S3 and S4, Supplementary Material online, respectively. At least 90% of placental UCEs are syntenic, as determined by flanking protein-coding genes in human and mouse genomes, allowing for differences in annotations and gene nomenclature between species (supplementary File S5, Supplementary Material online).

### Evolutionary History of UCEs

We traced the detection and conservation of the three sets of human UCE sequences during vertebrate and invertebrate evolution by mapping UCEs to >200 species (list of species supplementary table S5, Supplementary Material online) across animal phyla, compared with an equivalent sample of protein-coding sequences (CDSs).

**Fig. 3.**
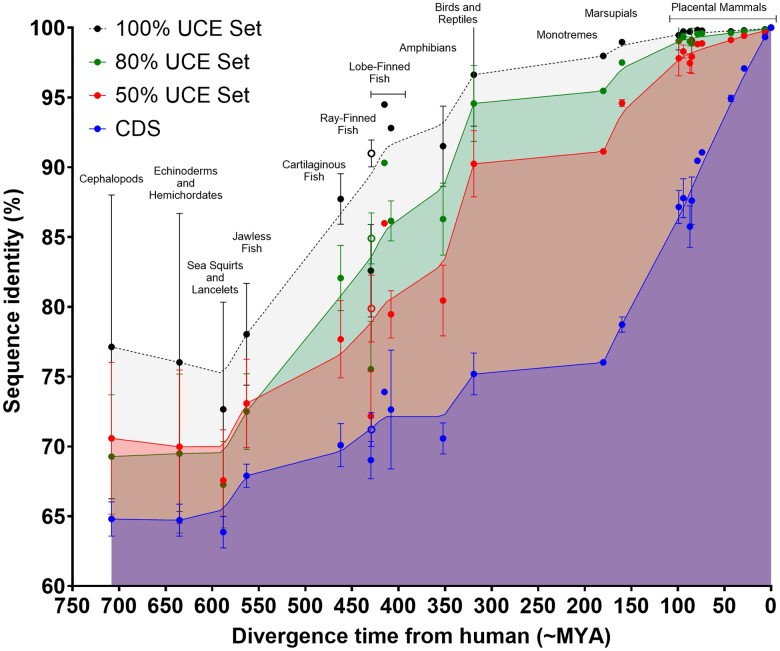
Sequence identity of human UCEs and CDSs throughout vertebrate evolution. The species plotted are listed in supplementary table S5, Supplementary Material online. Dots indicate the mean sequence identity (%) with human of the identified UCEs and CDSs of species in the group; vertical bars indicate range. Curves are a Lowess medium fitted curve. Open dots indicate non-teleost ray-finned fish. UCEs were required to occur in 50% (red), 80% (green), or 100% (black) of species in a set of 20 placental mammals, with ≥97% sequence identity over ≥100 bp.

We found that some UCEs could be mapped in species that diverged from humans between 550 and 670 Mya, with greater sequence identity than CDSs, but a comparatively low average sequence identity of ∼66% (Fig. 3 and supplementary fig. S6, Supplementary Material online). An obvious increase in sequence identity is observed in the jawless fish (based on the available genomes of extant species), especially in the most well conserved sequences, whereby sequence similarity increases to a greater extent than CDSs. A steep increase in UCE sequence identity is observed between the jawless fish and cartilaginous fish, where UCEs are ∼74% homologous to human UCEs, which is not observed in CDS sequences.

In the Osteichthyes (bony fish) (Trinajstic et al. 2022), which are divided into two major clades: the ray-finned fish (Actinopterygii) and lobe-finned fish (non-tetrapod Sarcopterygii), we found significant variation in sequence identity. Among the ray-finned fish, average UCE sequence identity was lower than the cartilaginous fish at ∼70.5%. However, maximum sequence identity (∼76%) increased, notably in the Polypteridae (reedfish and bichirs; see also Faircloth et al. 2013). The Polypteridae are currently resolved as the sister group to all other actinopterygians diverging at the actinopterygian stem (∼400 Mya) and retain a number of plesiomorphic traits including paired lungs whose development is similar to that in tetrapods (Tatsumi et al. 2016) and fleshy pectoral fins similar to those in lobe-finned fish, a lineage separate from the majority of the ray-finned fishes (see below).

We detected UCEs with a high average sequence identity (∼86%) in the coelacanth, a lobe-finned fish basal to the lungfish tetrapod lineage, containing a vestigial lung (Cupello et al. 2015), a primitive “hand” and a paired-fin movement pattern similar to tetrapod limb movement (Ahlberg 1989; Molnar et al. 2021). This especially holds for the most conserved set wherein UCEs were detected with an average of ∼94.5% identity to the human sequence, suggesting that strong purifying selection of the most conserved UCEs was in place by this point. Sequence identity of UCEs and CDS sequences are lower in the other sequenced non-tetrapod sarcopterygian, the lungfish (Fig. 3 and supplementary fig. S6, Supplementary Material online; see also Takezaki 2021), which is counterintuitive given recent analyses suggest that the lungfish are the closest extant relatives of the tetrapods (Takezaki and Nishihara 2017; Meyer et al. 2021). The seemingly lower sequence identity may be a technical artifact (due to differences in sequencing technology used and genome coverage, compounded by the enormous size of the lungfish genome), or may reflect a faster rate of molecular evolution in the lungfish lineage. Nonetheless, our data show that placental UCEs were present in the common ancestor of the Gnathostomata and were mostly established in sequence identity by the lobe-finned fishes.

UCEs in the amphibians, which diverged from other tetrapod lineages ∼370 Mya, are surprisingly variable, having an average of ∼80% sequence identity with human UCEs, lower than that of the coelacanth. Furthermore, the maximal sequence identity is higher in amphibians only in the more conserved sets, suggesting that some elements may have experienced reduced purifying selection during amphibian evolution.

In the birds and reptiles, UCEs can be mapped with ∼89% average sequence identity to human, and became near fixed. This is especially true of the more conserved sets, where UCEs have 94.5% to 95.5% sequence identity. There was little change in average UCE sequence identity between reptiles and monotremes, with <2% increase over ∼140 My, suggesting that UCEs became fixed in part before the split of reptiles and mammals. There is another increase in UCE sequence identity between monotremes and marsupials, except in the most conserved set, whose sequences are frozen. Unsurprisingly, we found that UCEs in placental mammals are almost identical to the human sequences, with an average sequence identity of 98% and a far slower evolutionary rate than coding sequences.

We observed a bimodal distribution in sequence identity of UCEs mapped to our species set (supplementary fig. S7, Supplementary Material online). The majority of UCEs that are detectable in lineages that arose before cartilaginous fishes mapped with less than ∼70% identity to the human sequence. We therefore chose a cutoff of 70% sequence identity to count the detection of UCEs during vertebrate evolution.

**Fig. 4.**
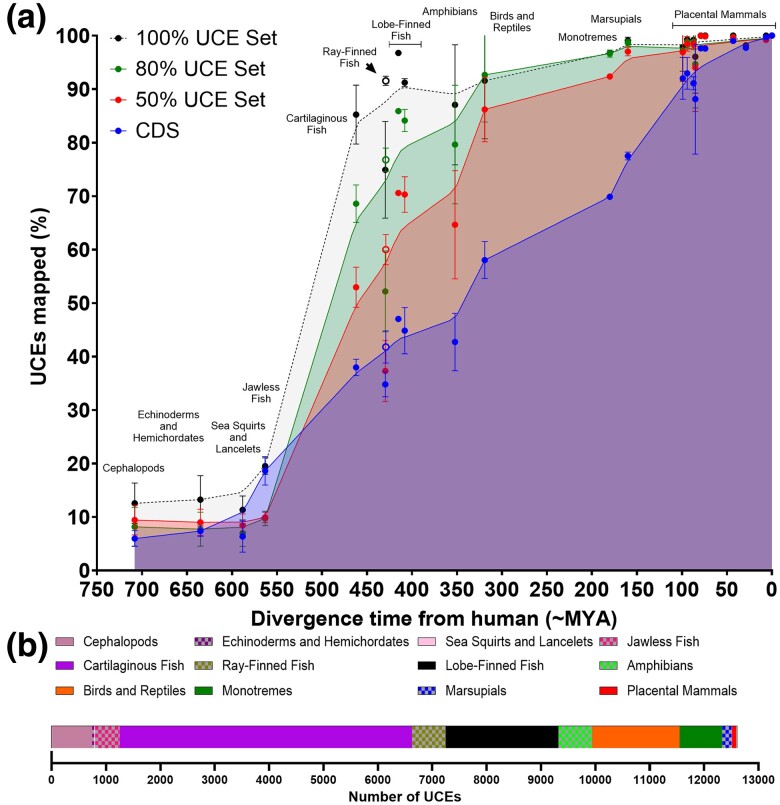
Emergence of UCEs during vertebrate evolution. a) Evolutionary history of UCE emergence. The species plotted are listed in supplementary table S5, Supplementary Material online. Dots indicate the mean % of UCEs or CDS mapped for species in the group; vertical bars indicate range. Curves are a Lowess medium fitted curve. Open dots indicate non-teleost ray-finned fish. UCEs were required to occur in 50% (red), 80% (green), or 100% (black) of species in a set of 20 placental mammals, with ≥97% sequence identity over ≥100 bp. b) Stacked bar chart of UCE emergence (50% set). UCEs were required to map in 50% of species in a clade.

Unsurprisingly, UCE detection followed a similar pattern to that of UCE sequence identity (Fig. 4a, b and supplementary fig. S8, Supplementary Material online). Approximately 10% of UCEs in all sets could be mapped with at least 70% sequence identity in species that diverged from humans more than 450 Mya. During the evolution of the jawed fishes, more than half of UCEs appeared (∼85% of the 100% mammalian species set), and ∼70% (∼95% in the 100% mammalian species set) were established prior to the evolution of lobe-finned fish and amphibians. There is a modest increase in the number of UCEs between lobe-finned fishes and the birds and reptiles, and little subsequent change during the evolution of the mammals, given the long evolutionary times between them. Although all three UCE sets exhibit similar patterns, the more stringent sets have greater sequence conservation at all time points, especially earlier time points, and a higher proportion appear earlier in vertebrate evolution.

Surprisingly, when we examined UCEs in the birds independently, we found ∼35,000 to 40,000 bird-centric UCEs (37,094 in chicken, supplementary File S6, Supplementary Material online) defined as ≥97% identical over ≥100 bp in ≥50% of 20 representative species (supplementary table S6, Supplementary Material online), and ∼30,000 with 100% sequence identity in at least half the species with larger average sizes than those in the placental mammals (supplementary fig. S9a, b, Supplementary Material online). This was not observed in reptiles with only ∼8,000 UCEs (supplementary File S7, Supplementary Material online) (defined as ≥97% identical over ≥100 bp in ≥50% of 20 representative species in supplementary table S7, Supplementary Material online). As the emu and chicken both contain almost all of these UCEs, bird UCEs are most likely not a function of the recent radiation of the bird lineage following the Cretaceous extinction 66 Mya (Brusatte Stephen et al. 2015; Field et al. 2018; Yu et al. 2021; Stiller et al. 2024). When mapped onto a subset of species (supplementary table S8, Supplementary Material online) the increases in sequence identity and UCE gain were more gradual than placental UCEs (supplementary fig. S9c, d, Supplementary Material online), suggesting that these bird UCEs are also conserved over great evolutionary distances.

### UCE Sequence Composition and Genomic Distribution

**Fig. 5.**
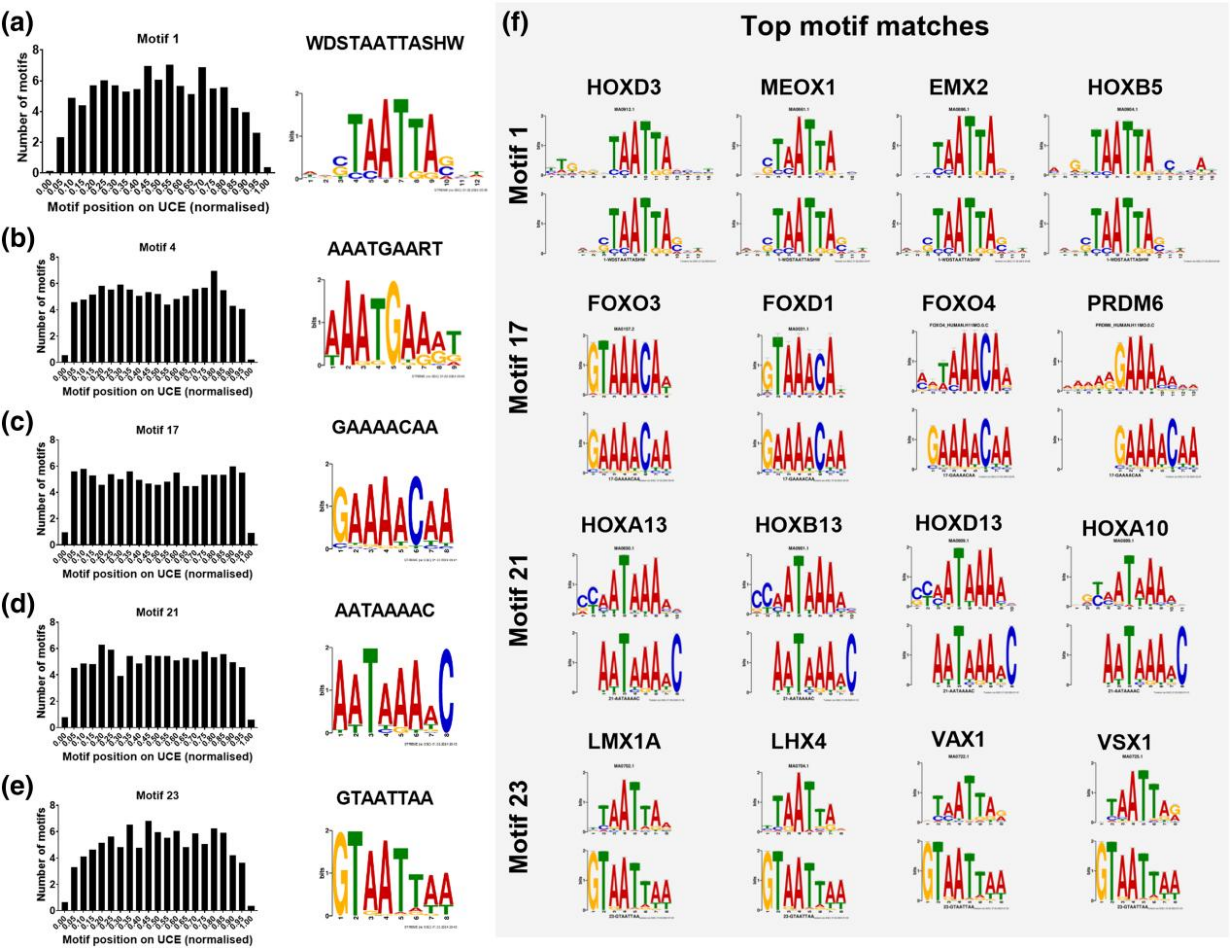
Common DNA motifs in UCEs. Five motifs were identified as both enriched in UCEs by STREME, and present in over 10% of UCEs. a to e) Distribution of motifs along UCEs. f) Four of these motifs aligned to transcription factor binding motifs using Tomtom.

As almost all UCEs are unique sequences (∼98.5%), we examined whether they exhibited similarities in sequence composition or genomic distribution. We examined sequence composition of human UCE sequences in two ways. First, we calculated the GC content of UCE sets, control CDSs and randomly shuffled sequences, and found that UCE sets (medians 36.7% to 37.8%) had a lower GC% than CDS sequences (median 51.9%) (supplementary fig. S10, Supplementary Material online). Second, using STREME from MEME Suite (Bailey 2021) we found 27 sequence motifs that are enriched in UCE sequences (supplementary File S8, Supplementary Material online; for 80% and 100% sets, see supplementary Files S9 and S10, Supplementary Material online). We focused on motifs present in >10% of UCEs (Fig. 5a to e), and plotted their normalized positions on UCEs, but found no obvious positional bias. We searched the motifs against two motif databases and found matches (15 to 216 matches per motif) for four of the five motifs (Fig. 5f, supplementary File S11, Supplementary Material online).

We also examined whether UCEs demonstrate any bias in their distribution either between or along human chromosomes. We found that UCEs are not equally distributed among chromosomes (adjusting for chromosome length) (supplementary fig. S11a, b, Supplementary Material online), nor evenly distributed along chromosomes, but occur in clusters (supplementary fig. S11c to e, Supplementary Material online), and are depleted on the short arms of almost half of human chromosomes (supplementary table S9, Supplementary Material online, chromosome Y excluded as some genome assemblies do not contain the male sex chromosome).

### Genomic Correlates of UCEs

**Fig. 6.**
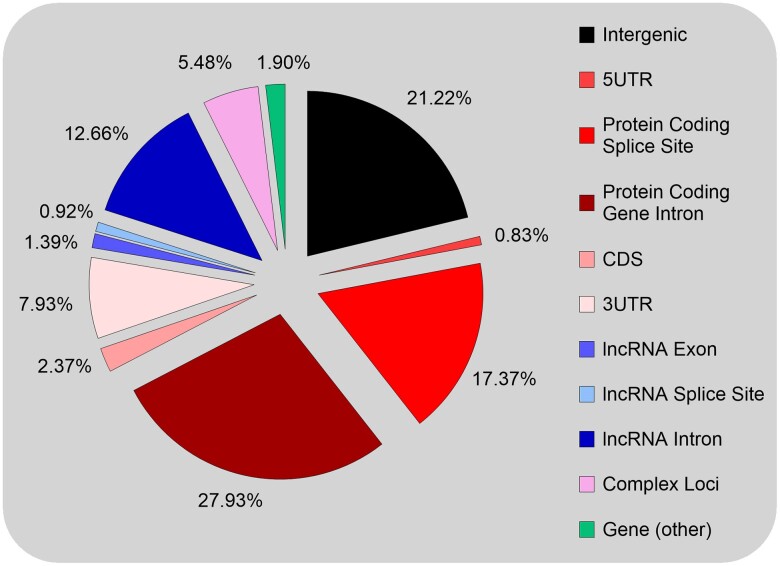
Genomic features containing mammalian ultraconserved elements. UCEs are predominantly located in intronic regions of protein-coding genes and lncRNAs, in intergenic regions, or at protein-coding gene intron-exon junctions. Complex loci include gene-intergenic junctions, and loci of overlapping protein-coding and lncRNA genes where the UCE cannot be designated to one gene type (i.e. the UCE is in the intronic region of both gene types, or overlapping exons/splice sites of both gene types).

We then examined genomic correlates of the UCE positions in the human genome. Of the 12,813 UCEs, 44 are located on random fragments in the human genome annotation and were excluded, leaving 12,769 UCEs for genomic correlate analysis. We found that the majority are either partially or wholly located in annotated genes from Ensembl (∼78.5% of all UCEs, Fig. 6), with ∼56% located within protein-coding genes, ∼15% within annotated long noncoding RNA (lncRNA) genes, and a small number occurring at more complex loci (∼5.5%) or in other types of genes (∼2%).

We confirmed that UCEs are predominantly located in nonprotein-coding sequences, with ∼63% occurring either in introns of protein-coding and lncRNA genes (∼41%) or in intergenic sequences (∼21%), noting that many “intergenic” regions may in fact be intronic given that many genes include distal 5′ exons that often overlap with upstream-annotated gene(s) (Willingham et al. 2006; Denoeud et al. 2007) and that genes are not discrete entities (Mattick 2003; Kapranov et al. 2005, 2007). Of total UCEs, ∼28% lie within protein-coding introns and ∼17.5% overlap intron-exon junctions, which is consistent with previous reports (Stephen et al. 2008), and ∼8% lie within 3′ UTRs. Few UCEs lie within 5′ UTRs (∼1%) or CDSs (∼2.5%, although 2,422 partially or fully overlap CDSs). Like protein-coding genes, lncRNA UCEs were predominantly intronic (∼12.5% of total UCEs), with few in exons (∼1.5%) or overlapping intron-exon junctions (∼1%). We found that this distribution generally held for all three UCE lists (supplementary fig. S12a to c, Supplementary Material online) but that a greater percent of UCEs in the 80% and 100% lists are intronic, with corresponding decreases in intergenic and intron-exon junction UCEs. A greater proportion of UCEs were also located in complex loci and other gene types in the more stringent sets.

### UCEs Are Concentrated in Genes Involved in Neuronal and Skeletomuscular Development

**Fig. 7.**
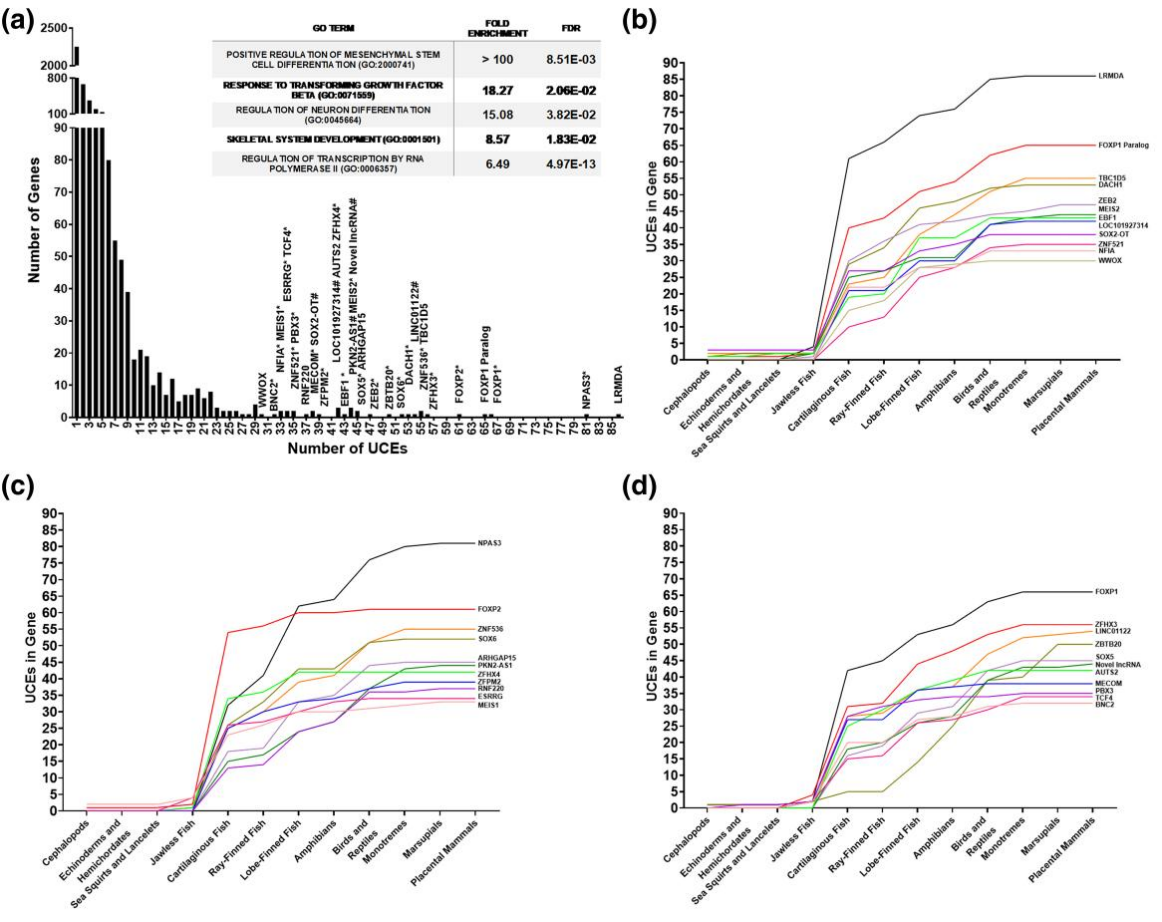
Genes containing UCEs. a) Most genes only contain one to ten UCEs, whilst very few contain ≥30. Genes with many UCEs are often transcription factors (*), whilst some are lncRNAs (#), are enriched for developmental and neurological functions (inset table). b to d) UCE gain in genes with ≥30 UCEs. Genes have acquired UCEs at different evolutionary time points and rates, whilst many have acquired large numbers of UCEs between the jawless and cartilaginous fish. Lines indicate individual genes. Genes have subdivided into three arbitrary sets for clarity.

As ∼80% of UCEs are present in annotated genes, we sought to identify which genes contained UCEs and the functions that these genes have. Whilst 10,061 of the 12,769 UCEs are located within annotated genes, only 4,049 contained UCEs, suggesting that UCEs are preferentially located in particular genes (Fig. 7a to d and supplementary File S12, Supplementary Material online). Supporting this, we found that 192 genes contained ≥10 UCEs, and 34 genes contained ≥30 UCEs. Genes with high numbers of UCEs were found to be enriched for Gene Ontologies related to regulation of differentiation, transcription, neuronal lineages and functions, and bone and muscle formation (Fig. 7a inset). Furthermore, genes have acquired UCEs at different stages of vertebrate evolution (Fig. 7b to d).

### UCEs Are Associated With Cis-Regulatory Elements

**Fig. 8.**
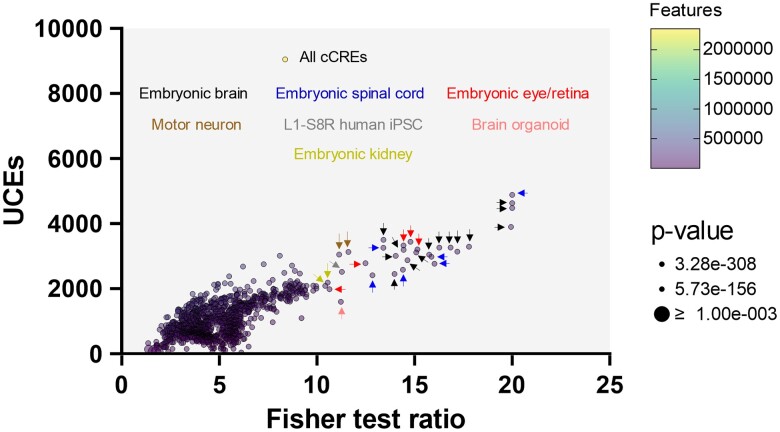
UCEs are enriched in candidate cis-regulatory elements (cCREs) from ENCODE datasets. A Fisher statistical test was used to test for enrichment of UCEs in cCREs. UCEs were enriched in many cCRE datasets, but especially those from embryonic CNS tissues. Color of arrows indicates tissue type of dataset. For plotting, Fisher test ratios > 20 were set to 20.

We examined whether UCEs are associated with regulatory domains in the genome. Using the regulatory build features of the hg38 genome from Ensembl and *bedtools*, we looked for overlap between UCEs and enhancers, promoters, transcription factor binding sites, CTCF-binding sites, and open-chromatin regions. There was significant overlap between UCE coordinates and most regulatory domains (supplementary fig. S13a, Supplementary Material online). We performed a more comprehensive assessment of UCE associations with regulatory domains by intersecting with ENCODE datasets for candidate cis-regulatory elements (cCREs) from thousands of tissues/cell samples (1,679 datasets, Fig. 8).

We also intersected UCEs with ENCODE transcription factor binding sites (TFBS, 29,890 datasets) and open chromatin (6,200) (supplementary fig. S13b, c, Supplementary Material online) and found further support for UCE association with cis-regulatory elements, especially in central nervous system (CNS) tissues.

### UCE Loci Are Expressed in Many Tissues and Are Developmentally Regulated, but Developmental Expression Is Not Evolutionarily Conserved

**Fig. 9.**
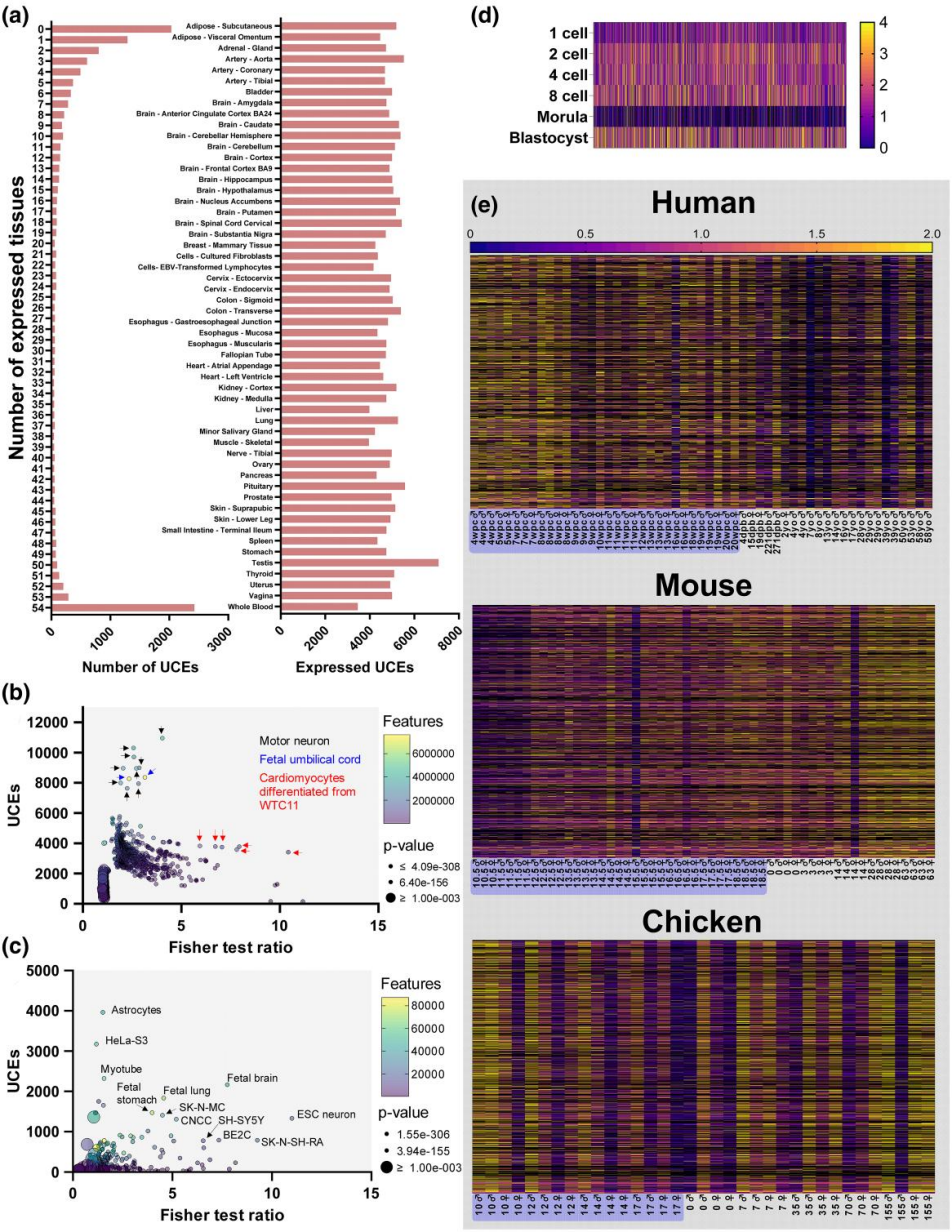
Expression of UCEs in human RNA-sequencing data. a) Expression of UCE loci in GTEx datasets. Number of tissues expressing each UCE (left). Number of UCEs expressed in each cell/tissue type (right). b) Enrichment of UCEs in ENCODE RNA-sequencing datasets. Arrows indicate tissue type. c) Enrichment of UCEs in enhancers from Enhancer Atlas. d) Expression of UCEs in early human embryonic development. Expression is presented as a fold change relative to average sample TPM. Only UCEs expressed in >10 samples are graphed. e) Forebrain/cortex expression of UCE loci in human, mouse, and chicken. Expression is presented as a fold change relative to average sample TPM. Sample names highlighted blue are embryonic time points.

Given the associations of UCEs with annotated genes and regulatory regions, especially enhancers, we reasoned that UCE loci may be producing functional RNAs, likely eRNAs. As no comprehensive characterization of expression from UCE loci has previously occurred, we analyzed the expression of human UCE loci across tissues, and developmental time points. We characterized tissue expression of UCE loci using publicly available BED files of the Genotype-Tissue Expression (GTEx) project RNA-sequencing data downloaded from the UCSC table browser (available at https://genome.ucsc.edu/cgi-bin/hgTables). We found that ∼2,500 UCEs were expressed in all 54 tissues investigated, whilst an almost equal number of UCEs were not expressed in any tissue (Fig. 9a). The remaining UCEs tended to be expressed in only a handful of tissues. There was no obvious bias of UCE expression to a particular tissue type, with most tissues expressing ∼4,000 to 5,000 UCE loci, except for the testis which expressed ∼7,500 UCE loci.

We also characterized expression of UCE loci using human RNA-sequencing data from ENCODE, by intersecting our UCE loci with expression data from >1,000 cell and tissue types (Fig. 9b). We found that motor neuron and fetal umbilical cord datasets expressed the greatest number of UCEs. As UCEs are associated with cis-regulatory elements and are transcribed, we reasoned that enhancers are produced from UCEs and intersected them with candidate enhancer loci from enhancer atlas (Gao and Qian 2020). We found that UCEs are associated with enhancers, especially those expressed in brain and fetal tissues (Fig. 9c).

To examine UCE loci expression during early embryonic development, we probed a recently published RNA-sequencing dataset that profiled the transcriptome from the one cell stage to the blastocyst (Torre et al. 2023). We found that ∼4,000 UCEs are robustly expressed in the human pre-implantation embryo, and that the expression of ∼1,400 are significantly different between two developmental stages (Fig. 9d), suggesting that at least some UCE loci encode developmentally regulated RNAs that are active in the earliest stages of embryo development.

To determine if expression profiles of UCEs are evolutionarily conserved, we utilized RNA-sequencing data from multiple species in seven different organs (forebrain, hindbrain, heart, kidney, liver, ovary, and testes) from 4 weeks post-conception until late adulthood (Cardoso-Moreira et al. 2019). We extracted the positions of human UCEs in the mouse and chicken genomes, and mapped the human, mouse, and chicken RNA-sequencing data to their respective genomes. Surprisingly, expression of loci across development was not consistent between species in forebrain/cortex (Fig. 9e), nor any other organ investigated (supplementary fig. S14a to f, Supplementary Material online). Whilst human expression was generally greater during development (brain, heart, kidney) or consistent (liver, ovary, testes), mouse expression was greater in the adult (except liver and ovary). Chicken expression was mixed with organs displaying consistent expression across lifespan (brain, heart, liver), greater expression during development (kidney), or during adulthood (ovary and testes).

## Discussion

Since their discovery in 2004, UCEs have remained an enigmatic characteristic of amniotic (and other) genomes. Originally defined as segments of the genome ≥ 200 bp with perfect sequence identity between human, rat, and mouse, UCEs (and similar highly conserved sequences) have since been defined in many animal clades including vertebrates such as mammals, birds, reptiles, amphibians, and fish (Bejerano et al. 2004; Crawford et al. 2012; McCormack et al. 2012, 2016; Faircloth et al. 2013; Das et al. 2023), invertebrates such as annelids, bivalves, gastropods, crustations, and insects (Glazov et al. 2005; Petersen et al. 2022), and even across diverse phyla (Ryu et al. 2012). These definitions have often been arbitrary, with formal definitions being constrained by limited genome availability, assembly and sequence quality, and software availability.

Given the far greater number of genomes currently available and improvements in genome quality thanks to improved sequencing technologies, we sought to revisit the definition of placental mammal UCEs, and examine their evolutionary distribution with greater resolution. To this end we introduce a new program, *dedUCE*, to identify UCEs in a user defined set of genomes. We validated this program against previously published lists of UCEs, and compared *dedUCE* against other available tools, *PHYLUCE* and *Progressive Cactus*. *dedUCE* makes significant improvements in ease of use, flexibility, accuracy, and speed over existing tools.

To define UCE sets, we selected definition and test set genomes spread across placental mammal orders to avoid phylogenetic bias. When not requiring UCEs to be detected in all species, there is the potential to introduce phylogenetic bias into UCE datasets if genomes are not carefully selected to avoid this. For example, if defining a ray-finned fish set of UCEs, random selection of available genomes could easily generate a UCE set that is heavily biased toward teleosts, given that they account for 1,488 of the 1,502 ray-finned fish reference genomes available from NCBI. We tested our UCE sets for phylogenetic bias by mapping them onto our definition and test genomes (supplementary fig. S4, Supplementary Material online), and found that we could map 97% to 100% of UCEs for all sets, with no obvious bias for or against any particular clade.

Using *dedUCE* we defined sets of UCEs in placental mammals as sequences with ≥97% sequence identity over at least 100 bp in 50%, 80%, or 100% of definition set species and mapped these sets to over 200 animal species to examine the evolutionary history of UCE sequences at high resolution. Our previous understanding of mammalian UCEs proposed that sequences rapidly evolved under strong positive selection during tetrapod evolution with widespread genome acquisition and expansion of UCEs, followed by strong negative selection in the amniotes (Stephen et al. 2008).

Our data suggest that placental mammal UCEs were under strong purifying selection earlier than originally proposed. Many UCEs appear to have been acquired after divergence of the Gnathostomata common ancestor from the jawless fish ∼560 Mya, but before divergence of the cartilaginous fish from the Osteichthyes ∼460 Mya, an evolutionary stage that saw the appearance of the jaw and paired limbs, and reproductive internal fertilization during vertebrate evolution. Our data support the conclusions of (Wang et al. 2008), that many UCEs were already present in the jawed vertebrate ancestor, however we suggest this number to be at least ∼50% of UCEs and find that only ∼10% were present before the jawed ancestor. Furthermore, we find that UCE sequences were largely established in early Sarcopterygii evolution—before divergence of the lobe-finned fishes from the tetrapods ∼415 Mya—and then became near fixed in the amniotes. These effects are even more obvious in the more highly conserved UCE sets, where UCEs appear to be near fixed since the early Sarcopterygii, suggesting that conservation of the different UCE sets may be driven by distinct evolutionary pressures. Together, our data suggest that the evolutionary pressures driving selection of many UCEs were present in the aquatic environments in the Cambrian–Devonian periods.

We confirmed that our UCE sets exhibit similar characteristics as those proposed previously, including that UCEs exhibit higher conservation in their core sequences than in their flanks, are predominantly located in intronic and intergenic regions of the genome, are associated with enhancers and promoters, and are located within important developmental genes (Bejerano et al. 2004; Stephen et al. 2008; Van Dam et al. 2021). After confirming that many UCEs occur in clusters within introns, we found that most genes that have acquired high numbers of UCEs have important neurodevelopmental, and/or skeletomuscular functions.

The gene with the highest number of UCEs (86), *LRMDA*, is required for the differentiation of melanocytes, which are derived from the neural crest, and present in many tissues. *NPAS3* (neuronal PAS domain protein 3, 81 UCEs) encodes a brain-enriched transcription factor required for hippocampal neurogenesis (Pieper et al. 2005). *FOXP1* and its paralogs (61 to 66 UCEs) encode transcription factors required for vertebrate brain development and function and display complex patterns of expression, suggesting that sophisticated regulatory mechanisms control its spatiotemporal expression (Bacon and Rappold 2012; Co et al. 2020). *ZFHX3* (also known as *ATBF1*, 56 UCEs) encodes a multiple homeodomain transcription factor that regulates neuronal and myogenic differentiation (Berry et al. 2001; Jung et al. 2005). *ZNF536* (55 UCEs) encodes a transcription factor that is expressed in the developing central nervous system and localized in the cerebral cortex, hippocampus, and hypothalamus (Qin et al. 2009). *TBC1D5* (55 UCEs) encodes a regulator of the retromer complex, which mediates retrograde transport and is crucial for synaptic function (Brodin and Shupliakov 2018). *Linc01122* (54 UCEs) is expressed primarily in the developing cerebral cortex (Scharf et al. 2019). *DACH1* (53 UCEs) encodes a chromatin-associated protein involved in digit development (Umair et al. 2021). *SOX6* (52 UCEs) is required for development of the central nervous system, chondrogenesis (cartilage and bone formation) and maintenance of cardiac and skeletal muscle cells (Hagiwara 2011; Stevanovic et al. 2021). *ZBTB20* (50 UCEs) affects dendritic and synaptic structure (Jones et al. 2018).

*ZEB2* (47 UCEs) is a key developmental regulator of the central nervous system, with multiple associated enhancers that drive expression patterns to specific brain regions (Bar Yaacov et al. 2019). *SOX5* (45 UCEs) encodes a transcription factor involved in the regulation of chondrogenesis and the development of the nervous system, including post-mitotic control of the neuronal migration, molecular identity and subcortical axonal projections of neurons (Liu and Lefebvre 2015; Stevanovic et al. 2021). *ArhGAP15* (45 UCEs) is expressed in excitatory and inhibitory neurons of the hippocampus (Zamboni et al. 2016). *PKN2-AS1* (44 UCEs) is antisense to *PKN2*, which is required for neural tube development (Danno et al. 2017). *Meis2* (44 UCEs) is essential for cranial and cardiac neural crest development (Machon et al. 2015). *EBF1* (43 UCEs) encodes a transcription factor required for neuronal cell differentiation and EBF family members are known to have important roles in several aspects of vertebrate neurogenesis, including commitment, migration and differentiation (Green and Vetter 2011). *AUTS2* (42 UCEs) has been implicated in neurodevelopment, with several brain-specific enhancers, and is associated with numerous neurological disorders, including autism spectrum disorders, intellectual disability, and developmental delay (Oksenberg et al. 2013). *LOC101927314* (42 UCEs) specifies a brain-specific multi-exonic lncRNA (Fagerberg et al. 2014). Not much is known about *ZFHX4* (42 UCEs) except that it is involved in brain development and calcification of cartilage matrices (Eising et al. 2019; Nakamura et al. 2021). Clearly, clusters of UCEs have become entrenched in key neurodevelopmental and musculoskeletal genes during vertebrate evolution, which suggests that they may act as developmental regulators of these genes.

By intersecting our UCE loci with GTEx datasets, we found that all tissues expressed UCEs, with ∼1,500 to 2,000 UCEs being either tissue specific, ubiquitously expressed, or not detected in any tissue. Comparisons to Ensembl datasets confirmed UCE association with cis-regulatory elements, whilst intersection with ENCODE and Enhancer Atlas datasets highlighted their association with neurons, neural progenitor cells, and fetal CNS tissues, suggesting their importance in embryonic CNS development, which is consistent with previous experimental work on a small number of UCEs (Dickel et al. 2018). Furthermore, we found that ∼1/3 of human UCEs are robustly expressed during pre-implantation embryonic development, and that ∼1/4 of those are differentially expressed during this period. Confusingly, whilst we found that UCEs are dynamically expressed during CNS (and other organ) development in human, mouse, and chicken, the patterns of developmental expression differed between species.

Together, these data indicate that UCEs are most likely associated with developmental enhancers that regulate the complex developmental innovations associated with more complex motor and neurological systems in tetrapods. It is becoming evident that enhancers express lncRNAs in the cells in which they are active, which may be related to their mechanism of action (Mattick et al. 2023). Notably a substantial proportion of UCEs are located in the introns of lncRNAs, which may be an underestimate given that only a minority of lncRNAs are annotated in public databases (Mattick et al. 2023), may also be expressed from “intergenic” regions (Bartonicek et al. 2017) and/or have poor transcript models (Deveson et al. 2017, 2018). The reason why UCE primary sequences are so highly conserved, however, remains a mystery (Snetkova et al. 2022), as UCE enhancer function does not require perfect sequence conservation (Snetkova et al. 2021). Furthermore, we demonstrate that developmental timing of UCE loci is not conserved between species, at least in the organs investigated. One possibility is that UCEs (or RNAs derived from them) have multilateral targets and/or regulators, which makes sequence co-variation exponentially difficult, which has been proposed for Drosophila UCEs (Warnefors et al. 2016).

## Materials and Methods

### Genomes

This research includes computations using the computational cluster Katana supported by Research Technology Services at UNSW Sydney (https://doi.org/10.26190/669x-a286). Genomes were downloaded from the National Center for Biotechnology Information database (NCBI; available at https://www.ncbi.nlm.nih.gov/), Ensembl database (available at https://asia.ensembl.org/index.html), or University of California Santa Cruz database (UCSC; available at http://genome.ucsc.edu). A minority of genomes were downloaded from other sources (see supplementary tables S3 to S8, Supplementary Material online for all sources of genomes). Genomes were selected to span across orders from the clades investigated to avoid lineage-specific effects. Where applicable, genomes with coverage > 20×, or genomes sequenced using Sanger sequencing, were selected to ensure base call accuracy of the genome. Both Ensembl and NCBI human genome annotations were used depending on the analysis. The Ensembl human Gene Transfer Format (GTF) annotation for the GRCh38.p13 build (last updated 08-2021) was downloaded from the Ensembl FTP site (available at https://ftp.ensembl.org/pub/release-108/gtf/homo_sapiens/). The NCBI GRCh38.p13 GTF file (version 2.2, assembly GCF_000001405.39) was downloaded from the NCBI FTP site (available at https://ftp.ncbi.nlm.nih.gov/genomes/all/GCF/000/001/405/GCF_000001405.39_GRCh38.p13/).

### UCE Identification Using *dedUCE*

#### dedUCE

UCEs were identified using a new program, *dedUCE*. The principle of *dedUCE* is to progressively reduce the UCE search space to a minimum before any computationally expensive window-based operations are performed. For details of *dedUCE*, see supplementary File S1, Supplementary Material online, and the GitHub https://github.com/slimsuite/deduce. For all analyses using dedUCE, alignments were performed using minimap2.

#### Validation of *dedUCE* Against Previously Published UCEs

To validate that the *dedUCE* program correctly identifies UCEs, we replicated discovery of UCEs from published lists. We selected 25 datasets from four studies where the parameters for UCE identification for each dataset and the genome versions used were clearly defined (Bejerano et al. 2004; Derti et al. 2006; Stephen et al. 2008; Makunin et al. 2013). BED files were available for three of the studies, comprising 23 datasets. We replicated the parameters used in each study with *dedUCE*. All BED files were sorted and merged using *bedtools sort* and *bedtools merge* from *Bedtools* 2.27.1 (Quinlan 2014). dedUCE BED files for the Makunin et al. (2013) vertebrate datasets were converted from hg18 to hg19 using UCSC liftover (available at https://genome.ucsc.edu/cgi-bin/hgLiftOver), as performed in the original study. For the eutherian UCE set from Stephen et al. (2008), UCEs were combined and mapped to hg19 using *glsearch36* from the *FASTA* package (fasta/36.3.8g available at https://github.com/wrpearson/fasta36) and filtered for matches ≥95% sequence identity. Where possible, BED files from *dedUCE* were intersected with dataset BED files using *bedtools intersect* to identify any UCEs missed by *dedUCE* and any extra UCEs identified by *dedUCE*. The number of UCE bases in each dataset (nearest kb) was also calculated by summing the lengths of UCEs. Where no BED file was available, data were taken from the original publication.

#### Comparisons to Other Methods to Identify UCEs

To benchmark *dedUCE*, we compared UCE discovery to two popular methods, *PHYLUCE* (Faircloth 2016) and multi-alignment with sliding-window discovery using *Progressive Cactus* (Armstrong et al. 2020; Christmas et al. 2023). For *PHYLUCE*, we followed the tutorial for identifying UCE loci, with some modifications (available at https://phyluce.readthedocs.io/en/latest/tutorials/tutorial-4.html). We simulated single-end reads at 50× coverage with quality scores of 40 and aligned the data to the set reference genome (hg18) using *bowtie2* v2.4.2 (available at https://github.com/BenLangmead/bowtie2) in end-to-end mode, set to only output perfectly aligned reads. We generated and merged BED files for the alignments, and for human-dog-species comparisons, we used *bedtools multiinter* to intersect the BED files from the dog alignments with the alignments for the other species in the set. We then filtered for loci with alignments in both species over 100 bp in length and intersected the *PHYLUCE* loci with dataset loci using *bedtools intersect*.

For multi-alignment, we used *Progressive Cactus*, an updated multi-alignment program recently applied to UCE discovery (Christmas et al. 2023). We performed multi-alignments for the different datasets, extracted alignments containing all species, created BED files from the human positions, and intersected the multi-alignment positions with the UCE positions.

#### Placental Mammal UCEs

We selected 20 placental mammals (supplementary table S3, Supplementary Material online) as representative species across the placental mammal orders, with the human genome acting as a reference for downstream analysis. To define UCEs in placental mammals using *dedUCE*, we selected a core k-mer length of 50 bases, core k-mer mapping limit of 1,000 positions in a genome, core k-mer threshold of 50% of species, variable core k-mer minimum sequence identity (same as UCE minimum sequence identity for the run), minimum UCE size of 100 bases, and maximum number for UCE multi-mapping of 100 positions in a genome. We then used *bedtools merge* to combine any overlapping UCE locations in a genome.

To limit the number of UCEs missed by using the more time-efficient aligner, *minimap2*, we ran *dedUCE* at sequence identities ranging from 100% to 93% and added the locations from the stricter homologies in a cumulative manner. That is, UCEs at 97% have the UCE locations identified by *dedUCE* for 100%, 99%, 98%, and 97% sequence identity runs, with overlapping locations merged.

#### Bird and Reptile UCEs

We selected 20 bird species (supplementary table S6, Supplementary Material online) to use as representative species across the bird orders, with the chicken genome acting as a reference (as for human in the placental sets) for downstream analysis. We also selected 20 reptilian species (supplementary table S7, Supplementary Material online) for reptile UCEs. We applied the same settings in *dedUCE* as those used for placental mammal UCE identification.

### Mapping UCE and Other Sequences in ∼200 Species

The 12,813 human UCE sequences identified by *dedUCE* were extracted from the human genome using *bedtools getfasta* (*Bedtools* 2.27.1). Human sequences were aligned to 209 vertebrate and invertebrate genomes using *glsearch36* from the *FASTA* package (fasta/36.3.8g available at https://github.com/wrpearson/fasta36). For sequence identity, the alignment with the highest bit-score for each UCE was selected, and alignments < 30 bp in length or alignments with *e*-value > 0.01 were removed. For counting of UCE numbers, the alignment with the highest bit-score for each UCE was selected, and alignments < 30 bp in length, alignments with *e*-value > 0.01, or alignments with sequence identity < 70% were removed. Divergence times were derived from *TimeTree* (Kumar et al. 2017).

For CDS sequences, a BED file was created of all human CDSs, and filtered for sequences between 100 and 1300 bp, approximating the range of sizes of UCEs for comparison, and a random sample of sequences equal to the number of UCEs (12,813) was taken for alignment. CDS alignments for calculation of average sequence identity and counting were filtered as per UCE filtering.

Conserved bases in the human genome were selected using the phyloP (phylogenetic *P*-value) scores from the 100 way multiple alignment of vertebrates from UCSC (available at https://github.com/wrpearson/fasta36) (Pollard et al. 2010). The BigWig file was downloaded and used to create bedgraph files for each chromosome using the *bigWigToBedGraph* program (available at http://hgdownload.cse.ucsc.edu/admin/exe/linux.x86_64/). Bedgraphs were transformed into BED files and bases with phyloP scores > 0 were selected. BED files were then sorted, merged, and had UCE locations (50% set) and repeat elements (downloaded from the UCSC Table Browser available at https://genome.ucsc.edu/cgi-bin/hgTables) removed using *bedtools subtract*. Sequences with lengths < 100 bp were removed and a sample of sequences equal to the number of UCEs (12,813) was taken for alignment to the nonhuman genomes in the 20 placental mammal set.

The set of chicken UCEs, and an equal number of chicken CDS sequences were mapped to a subset of species (supplementary table S8, Supplementary Material online). Alignments were filtered as for human UCEs and CDSs.

### UCE Synteny

UCE synteny between human and mouse was determined by identifying the nearest upstream and downstream protein-coding gene. Mouse was selected because protein-coding gene homologs are well defined and gene names are generally consistent between human and mouse. Protein-coding gene locations were extracted from the NCBI GRCh38.p13 GTF file (version 2.2, NCBI assembly GCF_000001405.39) to create a BED file of human protein-coding genes. The nearest upstream and downstream protein-coding gene to a UCE was identified using *bedtools closest* (*Bedtools* 2.27.1), allowing for and including all ties. A mouse protein-coding gene BED file was also created from the NCBI GRCm39 GTF file (version 2.2, NCBI assembly GCF_000001635.27), and the nearest upstream and downstream protein-coding genes of UCEs were identified. For mouse UCEs for synteny, human UCEs were mapped to the mouse genome, and the alignment with the highest bit-score was selected for each UCE, requiring a minimum sequence identity of 70%. Syntenic UCEs were defined as UCEs where the human and mouse location had at least one matching protein-coding gene upstream or downstream. As UCEs are not strand specific, “upstream” and “downstream” are arbitrary, so UCEs were considered syntenic where human “upstream” genes matched mouse “downstream” genes—although in most cases these matched. As protein-coding gene names can be different between human and mouse, we manually curated some UCEs that our analysis suggested were not syntenic, especially if large blocks of UCEs were not syntenic. Large blocks of “non-syntenic” UCEs were often syntenic, but upstream and downstream genes had different names to their mouse orthologs, or the most immediate upstream and downstream genes did not match between species, but the cluster of genes surrounding the UCE were similar. We did not manually validate all non-syntenic UCEs.

### UCE Sequence Composition and Genomic Distribution

GC% was measured using *bedtools nuc*. Differences in GC% were tested using a multiple comparisons corrected Mann–Whitney *U* test. Motif discovery on UCE sequences was performed using STREME from MEMEsuite (v5.5.1) with default settings. Motifs were compared to two motif databases using Tomtom from MEMEsuite using default settings (JASPAR2018_CORE_vertebrates_non-redundant.meme and HOCOMOCOv11_core_HUMAN_mono_meme_format.meme for database descriptions, see https://meme-suite.org/meme/db/motifs). Motifs were mapped to UCEs using FIMO from MEMEsuite, and motif start sites were normalized to UCE length to graph positional distribution of the motifs on the UCEs.

For UCE chromosomal distribution, we normalized the start site of each UCE to the size of the chromosome. We tested for enrichment of UCEs on short arms of chromosomes using a Chi-square test of the observed number of UCEs against an expected even distribution of UCEs along the chromosome.

### UCE Genomic Correlates

For genomic feature overlap, we created BED files from the Ensembl GTF file for human CDSs, 5′UTRs, 3′UTRs, lncRNA exons, protein-coding gene and lncRNA introns, intergenic regions, and other gene types (excluding lncRNAs and protein-coding genes). We intersected UCE BED files with feature BED files using *bedtools intersect*. We performed intersections against Ensembl regulatory elements, ENCODE datasets, and Enhancer Atlas datasets in the same way. Statistical enrichment was assessed using *bedtools fisher*.

To determine which genes contained UCEs, we used *bedtools closest* on UCE BED files with a BED file of all human genes from the Ensembl GTF file and filtered the output to include only UCE-gene overlaps, including all ties where they occurred. For mapping UCE expansion in genes, we collected all UCEs mapped in a clade with sequence identity of at least 70% with the human sequence.

### UCE Expression

To investigate tissue expression of UCE loci in human, we used *bigWigAverageOverBed* on GTEx bigwig files to get read coverage of UCE loci. We filtered loci based on coverage ≥ 10. For ENCODE RNA-sequencing datasets, we downloaded the datasets from the ENCODE experiment matrix (available at https://www.encodeproject.org/matrix/) and intersected all datasets with UCE loci using *bedtools*. We performed similar analyses using Enhancer Atlas datasets (available at http://www.enhanceratlas.org).

For developmental expression of UCE loci we downloaded pre-implantation embryo data (PRJNA787530) using the sra-toolkit, filtered read files using cutadapt, and aligned reads to the human genome (GRCh38, GCF_000001405.39_GRCh38.p13_genomic.fa, GCF_000001405.39_GRCh38.p13_genomic.gtf) using the STAR aligner (Dobin et al. 2013). Coverage of UCE coordinates was calculated using *bedtools coverageBed*, filtered for UCEs expressed in at least 10 of 71 samples, converted to TPM, and normalized to average UCE expression. Differences in embryonic stage UCE expression were tested by a Kruskal–Wallis Test followed by multiple comparisons corrected Mann–Whitney *U* tests.

For UCE loci expression during organ development, we downloaded bulk RNA expression data for human, mouse, and chicken organs from EMBL-EBI ArrayExpress (E-MTAB-6769 chicken, E-MTAB-6798 mouse, and E-MTAB-6814 human). Bam files were converted to fastq files using *bedtools bamToFastq*. Reads were aligned using STAR to the human (GRCh38, GCF_000001405.39_GRCh38.p13_genomic.fa, GCF_000001405.39_GRCh38.p13_genomic.gtf), mouse (GRCm39, GCF_000001635.27_GRCm39_genomic.fa, GCF_000001635.27_GRCm39_genomic.gtf), or chicken (GRCg7b, GCF_016699485.2_bGalGal1.mat.broiler.GRCg7b_genomic.fa, GCF_016699485.2_bGalGal1.mat.broiler.GRCg7b_genomic.gtf) genomes. Coverage of UCE coordinates was calculated using *bedtools coverageBed*, converted to TPM, and normalized to average UCE expression per species and organ.

## Supplementary Material

msae146_Supplementary_Data

## Data Availability

The primary data used in this study were obtained from genome databases downloaded from NCBI, Ensembl, or UCSC. Metadata are provided in the Supplementary Tables, Supplementary Material online and Supplementary Files, Supplementary Material online. All data are available from the authors upon request. Graphs were generated using *Graphpad Prism 9*. All software used in this study is in the public domain. *DedUCE* software repository is available on GitHub (https://github.com/slimsuite/deduce). References to other software repositories are given in the Materials and Methods.
