# Supplementary material for: The Evolution of Ultraconserved Elements in Vertebrates
Source: Mol Biol Evol. 2024 Jul 16;41(7):msae146. doi: 10.1093/molbev/msae146 (PMC11276968; doi:10.1093/molbev/msae146)
Supplement: msae146_Supplementary_Data [file msae146_supplementary_data.zip › Supplementary Information Tables and Figures.pdf]

Supplementary Information

Supplementary files

1. DedUCE details and validation.
2. Human genome coordinates of UCEs identified in 50% of species, grouped by chromosome.
3. Human genome coordinates of UCEs identified in 80% of species, grouped by chromosome.
4. Human genome coordinates of UCEs identified in 100% of species, grouped by chromosome.
5. Human and mouse syntenic UCEs.
6. Chicken genome coordinates of UCEs identified in 50% of species, grouped by chromosome.
7. Chicken coordinates of UCEs identified in 50% of species, grouped by chromosome.
8. STREME motifs 50% UCE set.
9. STREME motifs 80% UCE set.
10. STREME motifs 100% UCE set.
11. Tomtom 50% UCE set.
12. Human genes containing UCEs.

Supplementary Tables

Table S1: Validation of *dedUCE* against published UCE datasets.

Dataset	*Genomes in dataset*	*Number of UCEs in dataset*	*Number of UCEs identified by dedUCE*	*Percent of UCEs correctly identified by dedUCE*	*UCEs missed by dedUCE*	*Extra UCEs identified by dedUCE*	**UCE bases in dataset (nearest kb)**	**UCE bases dedUCE (nearest kb)**
Bejerano et al., (2004) H-M-R	Human (hg17) Mouse (mm6) Rat (rn3)	481	500	100	0	19	126	131
Derti et al., (2006) H-M-D	Human (hg17) Mouse (mm6) Dog (canFam1)	510	515	99.8	1	16	134	138
Derti et al., (2006) H-C	Human (hg17) Chicken (galGal2)	427	436	100	0	10	112	116
Derti et al., (2006) Combined dataset	HMR, HMD, and HC datasets	896	922	99.9	1	28	240	249
Makunin et al., (2013) H-D-Mouse	Human (hg18) Dog (canFam2) Mouse (mm8)	5257	5375	99.8	11	140	749	766
Makunin et al., (2013) H-D-Cow	Human (hg18) Dog (canFam2) Cow (bosTau3)	10984	11062	98.9	119	229	1638	1654
Makunin et al., (2013) H-D-Opossum	Human (hg18) Dog (canFam2) Opossum (monDom4)	4031	4124	99.8	8	111	596	609
Makunin et al., (2013) H-D-Platypus	Human (hg18) Dog (canFam2) Platypus (ornAna1)	2677	2539	98.4	42	174	392	374
Makunin et al., (2013) H-D-Chicken	Human (hg18) Dog (canFam2) Chicken (galGal3)	2481	2544	99.6	9	78	368	378

Makunin et al., (2013) H-D-Lizard	Human (hg18) Dog (canFam2) Lizard (anoCar1)	1285	1389	98.1	25	131	185	200
Makunin et al., (2013) H-D-Frog	Human (hg18) Dog (canFam2) Frog (xenTro2)	397	388	96.2	15	8	52	51
Makunin et al., (2013) H-D-Zebrafish	Human (hg18) Dog (canFam2) Zebrafish (danRer4)	22	33	100	0	11	3	4
Makunin et al., (2013) H-D-Fugu	Human (hg18) Dog (canFam2) Fugu (fr2)	20	20	100	0	0	2	2
Makunin et al., (2013) Combined H-D dataset	Combined dataset of all H-D-Species	12561	12751	99.3	89	319	1934	1966
Stephen et al., (2008) Eutherian dataset	Eutherian set	13736	14766	NA	NA	NA	2131	2258
Stephen et al., (2008) Fish dataset	Fish set	43	45	NA	NA	NA	5	5
Makunin et al., (2013) Mel-Ere-Yak	Drosophila Melanogaster (dm3) Drosophila Erecta (droEre2) Drosophila Yakuba (droYak2)	19232	20376	99	188	1351	2394	2630
Makunin et al., (2013) Mel-Ere-Ana	Drosophila Melanogaster (dm3), Drosophila Erecta (droEre2), Drosophila Ananassae (dana_r1.3)	1585	1620	97.5	40	75	186	192
Makunin et al., (2013) Mel-Ere-Pse	Drosophila Melanogaster (dm3), Drosophila Erecta (droEre2), Drosophila	695	709	98.6	10	24	81	83

	Pseudoobscura (dp4)							
Makunin et al., (2013) Mel-Ere-Wil	Drosophila Melanogaster (dm3), Drosophila Erecta (droEre2), Drosophila Willistoni (droWil1)	250	264	99.2	2	16	29	31
Makunin et al., (2013) Mel-Ere-Moj	Drosophila Melanogaster (dm3), Drosophila Erecta (droEre2), Drosophila Mojavensis (droMoj3)	217	236	99.1	2	21	26	30
Makunin et al., (2013) Mel-Ere-Vir	Drosophila Melanogaster (dm3), Drosophila Erecta (droEre2), Drosophila Virilis (droVir3)	221	232	98.2	4	15	26	27
Makunin et al., (2013) Mel-Ere-Gri	Drosophila Melanogaster (dm3), Drosophila Erecta (droEre2), Drosophila Grimshawi (droGri2)	213	226	99.5	1	14	25	27
Makunin et al., (2013) Combined Drosophila dataset	Combined dataset of all Mel-Ere-Species	19438	20579	99	193	1352	2421	2590
Makunin et al., (2013) Combined Sophophora dataset	Combined dataset of 4 comparisons Mel-Ere-Ana Mel-Ere-Pse Mel-Yak-Ana Mel-Yak-Pse	2126	2212	97.7	48	134	249	263

Table S2: Comparison of *dedUCE* against PHYLUCE and Progressive Cactus.

Dataset	***Dataset UCEs***	***Program***	***UCEs identified***	***UCEs % identified***	***UCEs missed***	***CPU time (h:m:s)***	***Memory used (kb)***
Makunin et al., (2013) H-D-Mouse	5257	dedUCE	5246	99.8	11	2:51:35	1.3E+08
		PHYLUCE	4817	91.6	440	10:05:54	1.3E+08
		Progressive Cactus	N/A	N/A	N/A	Not complete in 200hrs	N/A
Makunin et al., (2013) H-D-Cow	10984	dedUCE	10865	98.9	119	2:50:53	1.3E+08
		PHYLUCE	9998	91	986	8:55:24	1.3E+08
		Progressive Cactus	10836	98.7	148	2648:24:28	2.8E+08
Makunin et al., (2013) H-D-Opossum	4031	dedUCE	4023	99.8	8	2:23:14	1.3E+08
		PHYLUCE	3763	93.4	268	11:00:36	1.3E+08
		Progressive Cactus	3976	98.6	55	3685:10:43	2.7E+08
Makunin et al., (2013) H-D-Platypus	2677	dedUCE	2635	98.4	42	1:48:57	1.3E+08
		PHYLUCE	2492	93.1	185	8:03:19	1.3E+08
		Progressive Cactus	2610	97.5	67	2438:52:35	2.1E+08
Makunin et al., (2013) H-D-Chicken	2481	dedUCE	2472	99.6	9	1:40:10	1.2E+08
		PHYLUCE	2359	95.1	122	7:02:30	1.3E+08
		Progressive Cactus	2384	96.1	97	2473:31:26	2.2E+08
Makunin et al., (2013) H-D-Lizard	1285	dedUCE	1260	98.1	25	1:42:17	1.3E+08
		PHYLUCE	1211	94.2	74	7:45:34	1.3E+08
		Progressive Cactus	1241	96.6	44	1971:18:16	1.4E+08
Makunin et al., (2013) H-D-Frog	397	dedUCE	382	96.2	15	2:01:27	1.3E+08
		PHYLUCE	372	93.7	25	7:27:23	1.3E+08
		Progressive Cactus	361	90.9	36	2040:32:56	1.5E+08
Makunin et al., (2013) H-D-Zebrafish	22	dedUCE	22	100	0	2:05:19	1.3E+08
		PHYLUCE	19	86.4	3	7:55:25	1.3E+08
		Progressive Cactus	16	72.7	6	2000:14:01	1.4E+08
Makunin et al., (2013) H-D-Fugu	20	dedUCE	20	100	0	1:46:23	1.3E+08
		PHYLUCE	18	90	2	6:15:46	1.3E+08
		Progressive Cactus	13	65	7	1361:55:08	1.4E+08
Makunin et al., (2013) Combined H-D dataset	12561	dedUCE	12472	99.3	89	N/A	N/A
		PHYLUCE	11490	91.5	1071	N/A	N/A
		Progressive Cactus	N/A	N/A	N/A	N/A	N/A

Table S3: Genomes of the 20 placental mammals used for *dedUCE* testing and UCE identification.

Species	*Database*	*Genome Assembly*	*Size (Gb)*	*Coverage (Fold)*	*Order*	**Timetree Human Divergence Time Median**
Human	NCBI	GRCh38.p13	3.1	N/A	Primate	0
Marmoset	Ensembl	ASM275486v1	2.85	60	Primate	43
Sunda Flying Lemur	NCBI	GalVar_v2_BIUU_UCD	3.35	35.5	Dermoptera	79
Rabbit	NCBI	OryCun3.0	2.78	40	Lagomorpha	87
Mouse	NCBI	GRCm39	2.73	N/A	Rodentia	87
Chinese Tree Shrew	NCBI	TupChi_1.0	2.85	80	Scandentia	85
Cat	Ensembl	Felis_catus_9.0	2.52	72	Carnivora	94
Cow	Ensembl	ARS-UCD1.2	2.72	80	Cetartiodactyla	94
Large Flying Fox	NCBI	Pvam_2.0	2.2	188	Chiroptera	94
Common Shrew	NCBI	SorAra2.0	2.42	120	Eulipotyphla	94
Horse	Ensembl	EquCab3.0	2.51	88	Perissodactyla	94
Malayan Pangolin	NCBI	YNU_ManJav_2.0	2.44	411.8	Pholidota	94
Aardvark	NCBI	OryAfe_v1_BIUU	4.69	35.2	Tubulidentata	99
Small Madagascar Hedgehog	NCBI	ASM31398v2	2.95	78	Afrosoricida	99
Cape Rock Hyrax	NCBI	ProCapCap_v2_BIUU_UCD	3.9	23.3	Hyracoidea	99
Cape Elephant Shrew	NCBI	EleEdw1.0	3.84	62	Macroscelidea	99
Asian Elephant	NCBI	ASM1433276v1	3.13	94.4	Proboscidea	99
Armadillo	Ensembl	Dasnov3.0	3.63	6	Cingulata	99
Southern Two-Toed Sloth	NCBI	mChoDid1.pri	3.21	56.84	Pilosa	99
Florida Manatee	NCBI	TriManLat1.0	3.1	150	Sirenia	99

Table S4: Genomes of the 2nd 20 placental mammals set used for UCE set validation.

Species	***Database***	***Genome Assembly***	***Size (Gb)***	***Coverage (Fold)***	***Order***	***Timetree Human Divergence Time Median***
Chimpanzee	Ensembl	Pan_tro_3.0	3.23	55	Primate	6.4
Greater Bamboo Lemur	Ensembl	Prosim_1.0	2.41	152.7	Primate	74
American Pika	NCBI	OchPri4.0	2.23	23.68	Lagomorpha	87
Deer Mouse	NCBI	HU_Pman_2.1.3	2.51	115	Rodentia	87
Rat	NCBI	mRatBN7.2	2.65	92	Rodentia	87
Large Tree Shrew	NCBI	TupTan_v1_BIUU	3.65	25	Scandentia	85
Dog	NCBI	ROS_Cfam_1.0	2.4	56.5	Carnivora	94
Blue Whale	NCBI	mBalMus1.pri.v3	2.37	51.16	Cetartiodactyla	94
Jamaican Fruit-Eating Bat	NCBI	WHU_Ajam_v2	2.21	202	Chiroptera	94
Gracile Shrew Mole	NCBI	UroGra_v1_BIUU	2.11	54.2	Eulipotyphla	94
Western European Hedgehog	NCBI	EriEur2.0	2.72	79	Eulipotyphla	94
Donkey	NCBI	ASM1607732v2	2.43	211	Perissodactyla	94
Greater Indian Rhinoceros	NCBI	ASM1902286v1	2.52	70	Perissodactyla	94
Chinese Pangolin	NCBI	YNU_ManPten_2.0	2.4	281.6	Pholidota	94
Cape Golden Mole	NCBI	ChrAsi1.0	4.21	66	Afrosoricida	99
Yellow-Spotted Hyrax	NCBI	HetBruBak_v1_BIUU	3.62	24.4	Hyracoidea	99
African Elephant	NCBI	Loxafr3.0	3.2	7	Proboscidea	99
Screaming Hairy Armadillo	NCBI	ChaVel_v1_BIUU	5.34	22.9	Cingulata	99
Hoffman's Sloth	NCBI	C_hoffmanni-2.0.1	3.29	65	Pilosa	99
Dugong	NCBI	D_dugong_scaffold_01	2.62	64	Sirenia	99

Table S5: All genomes for UCE mapping.

Species	*Database*	*Genome Assembly*	*Size (Gb)*	*Coverage (Fold)*	*Clade*	*Timetree Human Divergence Time Median*
Human	NCBI	GRCh38.p13	3.10	N/A	Mammalia	0
Chimpanzee	Ensembl	Pan_tro_3.0	3.23	55	Mammalia	6.4
Rhesus Macaque	Ensembl	Mmul_10	2.97	66	Mammalia	28.8
Black-Handed Spider Monkey	NCBI	AteGeo_v1_BIUU	2.90	60	Mammalia	43
Marmoset	Ensembl	ASM275486v1	2.85	60	Mammalia	43
Brown Lemur	NCBI	EulFul_v1_BIUU	2.75	63.5	Mammalia	74
Greater Bamboo Lemur	Ensembl	Prosim_1.0	2.41	152.7	Mammalia	74
Sunda Flying Lemur	NCBI	GalVar_v2_BIUU_UCD	3.35	35.5	Mammalia	79
Chinese Tree Shrew	NCBI	TupChi_1.0	2.85	80	Mammalia	85
Large Tree Shrew	NCBI	TupTan_v1_BIUU	3.65	25	Mammalia	85
Northern Tree Shrew	Ensembl	tupBel1	3.67	2	Mammalia	85
American Pika	NCBI	OchPri4.0	2.23	23.68	Mammalia	87
Deer Mouse	NCBI	HU_Pman_2.1.3	2.51	115	Mammalia	87
Mouse	NCBI	GRCm39	2.73	N/A	Mammalia	87
Naked Mole-Rat	Ensembl	HetGla_1.0	3.04	90	Mammalia	87
Rabbit	NCBI	OryCun3.0	2.78	40	Mammalia	87
Rat	NCBI	mRatBN7.2	2.65	92	Mammalia	87
Blue Whale	NCBI	mBalMus1.pri.v3	2.37	51.16	Mammalia	94
Cat	Ensembl	Felis_catus_9.0	2.52	72	Mammalia	94
Chinese Pangolin	NCBI	YNU_ManPten_2.0	2.40	281.6	Mammalia	94
Common Shrew	NCBI	SorAra2.0	2.42	120	Mammalia	94
Cow	Ensembl	ARS-UCD1.2	2.72	80	Mammalia	94
Dog	NCBI	ROS_Cfam_1.0	2.40	56.5	Mammalia	94
Donkey	NCBI	ASM1607732v2	2.43	211	Mammalia	94
Eastern Mole	NCBI	ScaAqu_v1_BIUU	2.07	46.1	Mammalia	94
Gracile Shrew Mole	NCBI	UroGra_v1_BIUU	2.11	54.2	Mammalia	94
Greater Indian Rhinoceros	NCBI	ASM1902286v1	2.52	70	Mammalia	94
Horse	Ensembl	EquCab3.0	2.51	88	Mammalia	94
Jamaican Fruit-Eating Bat	NCBI	WHU_Ajam_v2	2.21	202	Mammalia	94
Large Flying Fox	NCBI	Pvam_2.0	2.20	188	Mammalia	94
Little Brown Bat	Ensembl	Myoluc2.0	2.03	7	Mammalia	94
Malayan Pangolin	NCBI	YNU_ManJav_2.0	2.44	411.8	Mammalia	94

Solenodon	NCBI	SolPar_v1_BIUU	2.11	29.3	Mammalia	94
Tree Pangolin	NCBI	ManTri_v1_BIUU	3.01	30.2	Mammalia	94
Western European Hedgehog	NCBI	EriEur2.0	2.72	79	Mammalia	94
Aardvark	NCBI	OryAfe_v1_BIUU	4.69	35.2	Mammalia	99
African Elephant	NCBI	Loxafr3.0	3.20	7	Mammalia	99
Armadillo	Ensembl	Dasnov3.0	3.63	6	Mammalia	99
Asian Elephant	NCBI	ASM1433276v1	3.13	94.4	Mammalia	99
Cape Elephant Shrew	NCBI	EleEdw1.0	3.84	62	Mammalia	99
Cape Golden Mole	NCBI	ChrAsi1.0	4.21	66	Mammalia	99
Cape Rock Hyrax	NCBI	ProCapCap_v2_BIUU_UCD	3.90	23.3	Mammalia	99
Dugong	NCBI	D_dugong_scaffold_01	2.62	64	Mammalia	99
Florida Manatee	NCBI	TriManLat1.0	3.10	150	Mammalia	99
Giant Anteater	NCBI	MyrTri_v1_BIUU	3.55	28.9	Mammalia	99
Hoffman's Sloth	NCBI	C_hoffmanni-2.0.1	3.29	65	Mammalia	99
Screaming Hairy Armadillo	NCBI	ChaVel_v1_BIUU	5.34	22.9	Mammalia	99
Small Madagascar Hedgehog	NCBI	ASM31398v2	2.95	78	Mammalia	99
Southern Three-Banded Armadillo	NCBI	TolMat_v1_BIUU	4.12	22.1	Mammalia	99
Southern Two-Toed Sloth	NCBI	mChoDid1.pri	3.21	56.84	Mammalia	99
Steller's Sea Cow	NCBI	H_Gigas_1.0	1.24	11	Mammalia	99
Yellow-Spotted Hyrax	NCBI	HetBruBak_v1_BIUU	3.62	24.4	Mammalia	99
Agile Gracile Opossum	NCBI	AgileGrace	3.70	100	Mammalia	160
Opossum	Ensembl	ASM229v1	3.60	6.8	Mammalia	160
Tasmanian Devil	Ensembl	mSarHar1.11	3.09	88	Mammalia	160
Wallaby	Ensembl	Meug_1.0	2.96	2	Mammalia	160
Echidna	NCBI	mTacAcu1.pri	2.21	58.29	Mammalia	180
Platypus	NCBI	mOrnAna1.pri.v4	1.86	58.8	Mammalia	180
American Flamingo	NCBI	bPhoRub2.pri	1.25	61.27	Aves	319
Anna's Hummingbird	NCBI	bCalAnn1_v1.p	1.06	54	Aves	319
Bald Eagle	NCBI	Haliaeetus_leucocephalus-4.0	1.18	103	Aves	319
Barn Owl	NCBI	T.alba_DEE_v4.0	1.25	103	Aves	319
Black-Tailed Trogon	NCBI	ASM1339927v1	1.11	69	Aves	319
Chicken	NCBI	bGalGal1.mat.broiler.GRCg7b	1.05	102.01	Aves	319

Common Cuckoo	NCBI	bCucCan1.pri	1.18	64.31	Aves	319
Duck	NCBI	ZJU1.0	1.19	143	Aves	319
Emperor Penguin	NCBI	ASM69914v1	1.25	60	Aves	319
Emu	NCBI	ASM1339679v1	1.20	71	Aves	319
Green Heron	NCBI	ASM1731025v1	1.19	53	Aves	319
Hooded Crane	NCBI	Gmonacha_1.0	1.18	67	Aves	319
Indigo-Banded Kingfisher	NCBI	ASM1340135v1	1.12	70	Aves	319
Kagu	NCBI	ASM1339809v1	1.12	49	Aves	319
Kakapo	NCBI	bStrHab1.2.pri	1.15	76.1	Aves	319
Lesser Kestrel	NCBI	bFalNau1.pat	1.22	104.34	Aves	319
Red-Faced Mousebird	NCBI	ASM1340125v1	1.11	89	Aves	319
Red-Legged Seriema	NCBI	bCarCri1.pri	1.22	50.93	Aves	319
Rock Pigeon	NCBI	Cliv_2.1	1.11	60	Aves	319
Zebra Finch	NCBI	bTaeGut1.4.pri	1.06	88.2	Aves	319
Agassiz's Desert Tortoise	NCBI	ASM289641v1	2.18	118	Reptilia	319
American Alligator	NCBI	ASM28112v4	2.16	156	Reptilia	319
Australian Saltwater Crocodile	Ensembl	CroPor_comp1	2.05	74	Reptilia	319
Burmese Python	NCBI	Python_molurus_bivittatus-5.0.2	1.44	20	Reptilia	319
Calyptommatus Sinebrachiatus	NCBI	HLcalSin1	1.75	167	Reptilia	319
Central Bearded Dragon	NCBI	pvi1.1	1.72	83	Reptilia	319
Chinese Alligator	NCBI	ASM45574v1	2.27	109	Reptilia	319
Common Snapping Turtle	NCBI	ASM1885937v1	2.26	125.81	Reptilia	319
Common Wall Lizard	NCBI	PodMur_1.0	1.51	100	Reptilia	319
Desert Horned Lizard	NCBI	MUOH_PhPlat_1.1	1.90	21053.74	Reptilia	319
Eastern Brown Snake	Ensembl	EBS10Xv2-PRI	1.59	73	Reptilia	319
Gekko Japonicus	NCBI	Gekko_japonicus_V1.1	2.49	95	Reptilia	319
Gharial	NCBI	GavGan_comp1	2.64	81	Reptilia	319
Green Sea Turtle	NCBI	rCheMyd1.pri.v2	2.13	60	Reptilia	319
Komodo Dragon	NCBI	ASM479886v1	1.51	144	Reptilia	319
Myanophis Thanlyinensis	NCBI	ASM1765603v1	1.32	81	Reptilia	319
Pitted-Shelled Turtle	NCBI	Carettochelys_insculpta-1.0	2.36	68	Reptilia	319
Tiger Rattlesnake	NCBI	ASM1654583v1	1.61	168	Reptilia	319
Tretioscincus Oriximinensis	NCBI	HLtreOri1	1.56	221	Reptilia	319
Tuatara	NCBI	ASM311381v1	4.27	127	Reptilia	319

Tuberculate Toadhead Turtle	NCBI	Mesoclemmys_tuberculata-1.0	2.03	73	Reptilia	319
Western Rat Snake	NCBI	UNIGE_PanObs_1.0	1.69	92	Reptilia	319
African Bullfrog	NCBI	Pads_1.0	1.56	189	Amphibia	352
African Clawed Frog	NCBI	Xenopus_laevis_v10.1	2.74	28.5	Amphibia	352
American Bullfrog	NCBI	RCv2.1	6.25	66	Amphibia	352
Asiatic Toad	NCBI	ASM1485885v1	4.55	103	Amphibia	352
Common Coquí	NCBI	UCB_Ecoq_1.0	2.79	240	Amphibia	352
Common Frog	NCBI	aRanTem1.1	4.11	63	Amphibia	352
Common Toad	NCBI	aBufBuf1.1	5.04	64	Amphibia	352
Congo Dwarf Clawed Frog	NCBI	UCB_Hboe_1.0	3.21	110	Amphibia	352
Couch's Spadefoot Toad	NCBI	usc_Scouchii_0.1	0.48	25	Amphibia	352
Eastern Spadefoot Toad	NCBI	usc_Sholbrookii_0.1	0.71	39	Amphibia	352
Gabon Caecilian	NCBI	aGeoSer1.2	3.78	67	Amphibia	352
Japanese Wrinkled Frog	NCBI	Glandirana_rugosa_assembly_1.0	7.63	154	Amphibia	352
Marine Toad	NCBI	RM170330	2.55	141	Amphibia	352
Mexican Spadefoot Toad	NCBI	usc_Smult_1.0	1.08	21	Amphibia	352
Microcaecilia Unicolor	NCBI	aMicUni1.2	4.69	53	Amphibia	352
Nanorana Parkeri	NCBI	ASM93562v1	2.05	83	Amphibia	352
Ornate Burrowing Frog	NCBI	ASM1661782v1	1.07	140	Amphibia	352
Plains Spadefoot Toad	NCBI	usc_Sbombifrons_0.1	0.77	159	Amphibia	352
Sabana Surinam Toad	NCBI	UCB_Ppar_1.0	1.37	15	Amphibia	352
Spiny Toad	Ensembl	ASM966780v1	3.55	80.3	Amphibia	352
Strawberry Poison Frog	NCBI	UCB_Opum_1.0	3.49	136	Amphibia	352
Tropical Clawed Frog	NCBI	UCB_Xtro_10.0	1.45	111.5	Amphibia	352
Tungara Frog	NCBI	UCB_Epus_1.0	2.56	103	Amphibia	352
Two-Lined Caecilian	NCBI	aRhiBiv1.2	5.32	43	Amphibia	352
Yunnan Mustache Toad	NCBI	ASM1899414v1	3.54	78	Amphibia	352
Australian Lungfish	NCBI	neoFor_v3	34.56	30	Sarcopterygii	408
West African Lungfish	NCBI	PAN1.0	40.05	50	Sarcopterygii	408
Coelacanth	NCBI	LatCha_J1.0	2.74	299.48	Sarcopterygii	415
Alligator Gar	NCBI	BGI_Aspa_1.0	1.06	88	Actinopterygii	429
Angler	NCBI	BF2_Nord	0.75	150	Actinopterygii	429
Aplochiton Taeniatus	NCBI	fAplTae1.pri	0.46	60	Actinopterygii	429
Arapaima	NCBI	ASM784422v1	0.67	58.6	Actinopterygii	429
Atlantic Cod	NCBI	gadMor3.0	0.67	83	Actinopterygii	429

Atlantic Herring	NCBI	Ch_v2.0.2	0.79	75	Actinopterygii	429
Bowfin	NCBI	AmiCal1	0.83	22887.41	Actinopterygii	429
Common Carp	NCBI	ASM1834038v1	1.68	184.8	Actinopterygii	429
Eulachon	NCBI	Tpac_1.0	0.42	210	Actinopterygii	429
European Perch	NCBI	GENO_Pfluv_1.0	0.95	135	Actinopterygii	429
Fugu	NCBI	fTakRub1.3	0.38	83	Actinopterygii	429
Glacier Lanternfish	NCBI	ASM90032337v1	0.68	19	Actinopterygii	429
Gray Bichir	NCBI	ASM1683550v1	3.67	64	Actinopterygii	429
Jewelled Blenny	NCBI	fSalaFa1.1	0.80	96	Actinopterygii	429
Lake Trout	NCBI	SaNama_1.0	2.35	89	Actinopterygii	429
Live Sharksucker	NCBI	fEcheNa1.2	0.54	62	Actinopterygii	429
Nile Bichir	NCBI	ASM2640214v1	3.77	8427.66	Actinopterygii	429
Paddlefish	NCBI	ASM1765450v1	1.54	30	Actinopterygii	429
Reedfish	NCBI	fErpCal1.3	3.61	58	Actinopterygii	429
Sand Roller	NCBI	ASM90030228v1	0.46	35	Actinopterygii	429
Spotted Gar	NCBI	LepOcu1	0.95	677.5	Actinopterygii	429
Sterlet	NCBI	ASM1064508v1	1.83	42	Actinopterygii	429
Striped Catfish	NCBI	GENO_Phyp_1.0	0.76	147	Actinopterygii	429
Tarpon	NCBI	MATL_1.0	0.99	128.5	Actinopterygii	429
Yellowfin Tuna	NCBI	fThuAlb1.1	0.79	45	Actinopterygii	429
Yellowstripe Goby	NCBI	ASM1673593v1	1.00	164	Actinopterygii	429
Zebrafish	NCBI	GRCz11	1.37	N/A	Actinopterygii	429
Brownbanded Bambooshark	NCBI	Cpunctatum_v1.0	3.38	45	Chondrichthyes	462
Cloudy Catshark	NCBI	Storazame_v1.0	4.47	68	Chondrichthyes	462
Elephant Shark	NCBI	IMCB_Cmil_1.0	0.99	68.84	Chondrichthyes	462
Great White Shark	NCBI	sCarCar2.pri	4.29	66.84	Chondrichthyes	462
Little Skate	NCBI	LER_WGS_1	1.56	26	Chondrichthyes	462
Smaller Spotted Catshark	NCBI	sScyCan1.2	4.22	63	Chondrichthyes	462
Small-Eyed Rabbitfish	NCBI	UP_Haf	1.11	35	Chondrichthyes	462
Smalltooth Sawfish	NCBI	sPriPec2.1.pri	2.27	61.91	Chondrichthyes	462
Thorny Skate	NCBI	sAmbRad1.1.pri	2.56	128.24	Chondrichthyes	462
Whale Shark	NCBI	RhiTyp_1.0	2.82	164	Chondrichthyes	462
Whitespotted Bambooshark	NCBI	ASM401019v1	3.56	280	Chondrichthyes	462
Arctic Lamprey	NCBI	IMCB_Ljap_1.0	1.07	87.8	Cyclostomata	563
Brown Hagfish	NCBI	Eptata_v1	2.52	250	Cyclostomata	563
Far Eastern Brook Lamprey	NCBI	ASM1570882v1	1.06	96.13	Cyclostomata	563
Inshore Hagfish	NCBI	Eburgeri_4.0	2.62	240	Cyclostomata	563

Pacific Lamprey	NCBI	ETRf_v1	0.93	43	Cyclostomata	563
Sea Lamprey	NCBI	kPetMar1.pri	1.09	62.36	Cyclostomata	563
Aplidium Turbinatum	NCBI	kaAplTurb1.1	0.61	65	Ascidiacea	588
Black Sea Squirt	NCBI	Phfumi_MTP2014	0.23	120	Ascidiacea	588
Botryllus Schlosseri	NCBI	356a-chromosome-assembly	0.58	400	Ascidiacea	588
Halocynthia Aurantium	NCBI	Haaura_MTP2014	0.13	250	Ascidiacea	588
Halocynthia Roretzi	NCBI	Harore_MTP2014	0.12	200	Ascidiacea	588
Pacific Transparent Sea Squirt	NCBI	ASM14926v1	0.59	13	Ascidiacea	588
Styela Clava	NCBI	ASM1312258v2	0.34	100	Ascidiacea	588
Vase Tunicate	NCBI	Cint(typeB-Plymouth)_1.0	0.18	677	Ascidiacea	588
Warty Sea Squirt	NCBI	Phmamm_MTP2014	0.23	78	Ascidiacea	588
Belcher's Lancelet	NCBI	Haploidv18h27	0.43	29.86	Leptocardii	588
European Lancelet	NCBI	BraLan3	0.47	146	Leptocardii	588
Florida Lancelet	NCBI	Bfl_VNyyK	0.51	10.32	Leptocardii	588
Bat Star	NCBI	Pmin_3.0	0.61	150	Asteroidea	635
Crown-of-Thorns Starfish	NCBI	PKT-COTS_1.0	0.40	533	Asteroidea	635
Octopus Starfish	NCBI	ASM2101432v1	0.56	116	Asteroidea	635
Purple Sea Star	NCBI	ASM1099431v2	0.40	212.8	Asteroidea	635
Anneissia Japonica	NCBI	ASM1163010v1	0.59	100	Crinoidea	635
Green Sea Urchin	NCBI	Lvar_3.0	0.87	130	Echinoidea	635
Hemicentrotus Pulcherrimus	NCBI	HpulGenome_v1	0.57	100	Echinoidea	635
Purple Sea Urchin	NCBI	Spur_5.0	0.92	123	Echinoidea	635
Ptychodera Flava	NCBI	ptychodera_flava version 1.0.14	1.23	167	Enteropneusta	635
Saccoglossus Kowalevskii	NCBI	Skow_1.1	0.78	7	Enteropneusta	635
Brown Rock Sea Cucumber	NCBI	ASM993650v2	1.07	112	Holothuroidea	635
Greenfish Sea Cucumber	NCBI	IOCAS_Schl_1.0	0.72	514	Holothuroidea	635
Japanese Sea Cucumber	NCBI	ASM275485v1	0.80	367	Holothuroidea	635
Warty Sea Cucumber	NCBI	Ppar_1.0	0.87	339	Holothuroidea	635
Spiny Brittle Star	NCBI	Ospi.un_1.0	2.76	275	Ophiuroidea	635
California Two-Spot Octopus	NCBI	Octopus_bimaculoides_v2_0	2.34	92	Cephalopoda	708
Chambered Nautilus	NCBI	ASM1838910v1	0.73	112	Cephalopoda	708
Common Octopus	NCBI	ASM395772v1	1.77	80	Cephalopoda	708

East Asian Common Octopus	NCBI	ASM634580v1	2.72	285	Cephalopoda	708
Giant Squid	NCBI	UP_Adux	2.69	126	Cephalopoda	708
Hawaiian Bobtail Squid	NCBI	PBRC_Esco_1.0	5.28	105	Cephalopoda	708
Longfin Inshore Squid	NCBI	UCB_Dpea_1	4.60	30	Cephalopoda	708
Pharaoh Cuttlefish	NCBI	SPHA2.0	4.79	88	Cephalopoda	708
Southern Blue-Ringed Octopus	NCBI	ASM1550113v1	4.01	20.5	Cephalopoda	708
Sparkling Enope	NCBI	Wsci_v1.0	0.65	24	Cephalopoda	708

Table S6: Genomes of the 20 birds used for UCE identification.

Species	*Database*	*Genome Assembly*	*Size (Gb)*	*Coverage (Fold)*	*Order*	*Timetree Chicken Divergence Time Median*
Chicken	NCBI	bGalGal1.mat.broiler.GRCg7b	1.05	102.01	Galliformes	0
Duck	NCBI	ZJU1.0	1.19	143	Anseriformes	83
Bald Eagle	NCBI	Haliaeetus_leucocephalus-4.0	1.18	103	Accipitriformes	91
Anna's Hummingbird	NCBI	bCalAnn1_v1.p	1.06	54	Apodiformes	91
Red-Legged Seriema	NCBI	bCarCri1.pri	1.22	50.93	Cariamiformes	91
Red-Faced Mousebird	NCBI	ASM1340125v1	1.11	89	Coliiformes	91
Rock Pigeon	NCBI	Cliv_2.1	1.11	60	Columbidae	91
Indigo-Banded Kingfisher	NCBI	ASM1340135v1	1.12	70	Coraciiformes	91
Common Cuckoo	NCBI	bCucCan1.pri	1.18	64.31	Cuculiformes	91
Kagu	NCBI	ASM1339809v1	1.12	49	Eurypygiformes	91
Lesser Kestrel	NCBI	bFalNau1.pat	1.22	104.34	Falconiformes	91
Hooded Crane	NCBI	Gmonacha_1.0	1.18	67	Gruiformes	91
Zebra Finch	NCBI	bTaeGut1.4.pri	1.06	88.2	Passeriformes	91
Green Heron	NCBI	ASM1731025v1	1.19	53	Pelecaniformes	91
American Flamingo	NCBI	bPhoRub2.pri	1.25	61.27	Phoenicopteriformes	91
Kakapo	NCBI	bStrHab1.2.pri	1.15	76.1	Psittaciformes	91
Emperor Penguin	NCBI	ASM69914v1	1.25	60	Sphenisciformes	91
Barn Owl	NCBI	T.alba_DEE_v4.0	1.25	103	Strigiformes	91
Black-Tailed Trogon	NCBI	ASM1339927v1	1.11	69	Trogoniformes	91
Emu	NCBI	ASM1339679v1	1.20	71	Casuariiformes	108

Table S7: Genomes of the 20 reptiles used for UCE identification.

Species	*Database*	*Genome Assembly*	*Size (Gb)*	*Coverage (Fold)*	*Order*	*Timetree Chicken Divergence Time Median*
Chicken	NCBI	bGalGal1. mat.broiler. GRCg7b	1.05	102.01	Galliformes	0
Lesser Kestrel	NCBI	bFalNau1. pat	1.22	104.34	Falconiformes	91
Zebra Finch	NCBI	bTaeGut1. 4.pri	1.06	88.2	Passeriformes	91
Emperor Penguin	NCBI	ASM69914 v1	1.25	60	Sphenisciformes	91
Emu	NCBI	ASM13396 79v1	1.20	71	Casuariiformes	108
American Alligator	NCBI	ASM28112 v4	2.16	156	Crocodilia	245
Australian Saltwater Crocodile	Ensembl	CroPor_co mp1	2.05	74	Crocodilia	245
Chinese Alligator	NCBI	ASM45574 v1	2.27	109	Crocodilia	245
Gharial	NCBI	GavGan_c omp1	2.64	81	Crocodilia	245
Agassiz's Desert Tortoise	NCBI	ASM28964 1v1	2.18	118	Testudines	261
Common Snapping Turtle	NCBI	ASM18859 37v1	2.26	125.81	Testudines	261
Green Sea Turtle	NCBI	rCheMyd1. pri.v2	2.13	60	Testudines	261
Pitted Shelled Turtle	NCBI	Carettoche lys_insculp ta-1.0	2.36	68	Testudines	261
Tuberculate Toadhead Turtle	NCBI	Mesoclem mys_tuber culata-1.0	2.03	73	Testudines	261
Tuatara	NCBI	ASM31138 1v1	4.27	127	Rhynchocephali a	280
Common Wall Lizard	NCBI	PodMur_1. 0	1.51	100	Squamata	280
Burmese Python	NCBI	Python_mo lurus_bivitt atus-5.0.2	1.44	20	Squamata	280
Komodo Dragon	NCBI	ASM47988 6v1	1.51	144	Squamata	280
Tiger Rattlesnake	NCBI	ASM16545 83v1	1.61	168	Squamata	280
Gekko Japonicus	NCBI	Gekko_jap onicus_V1. 1	2.49	95	Squamata	280

Table S8: Species subset used for bird UCE mapping.

Species	***Database***	***Genome Assembly***	***Size (Gb)***	***Coverage (Fold)***	***Clade***	***Timetree Chicken Divergence Time Median***
Chicken	NCBI	bGalGal1.mat.broiler.GRCg7b	1.05	102.01	Aves	0
Duck	NCBI	ZJU1.0	1.19	143	Aves	83
American Flamingo	NCBI	bPhoRub2.pri	1.25	61.27	Aves	91
Bald Eagle	NCBI	Haliaeetus_leucocephalus-4.0	1.18	103	Aves	91
Barn Owl	NCBI	T.alba_DEE_v4.0	1.25	103	Aves	91
Common Cuckoo	NCBI	bCucCan1.pri	1.18	64.31	Aves	91
Emperor Penguin	NCBI	ASM69914v1	1.25	60	Aves	91
Green Heron	NCBI	ASM1731025v1	1.19	53	Aves	91
Hooded Crane	NCBI	Gmonacha_1.0	1.18	67	Aves	91
Indigo-Banded Kingfisher	NCBI	ASM1340135v1	1.12	70	Aves	91
Kagu	NCBI	ASM1339809v1	1.12	49	Aves	91
Red-Faced Mousebird	NCBI	ASM1340125v1	1.11	89	Aves	91
Red-Legged Seriema	NCBI	bCarCri1.pri	1.22	50.93	Aves	91
Rock Pigeon	NCBI	Cliv_2.1	1.11	60	Aves	91
Emu	NCBI	ASM1339679v1	1.2	71	Aves	108
Australian Saltwater Crocodile	Ensembl	CroPor_comp1	2.05	74	Reptilia	245
Aardvark	NCBI	OryAfe_v1_BIUU	4.69	35.2	Mammalia	319
Echidna	NCBI	mTacAcu1.pri	2.21	58.29	Mammalia	319
Human	NCBI	GRCh38.p13	3.1	N/A	Mammalia	319
Tasmanian Devil	Ensembl	mSarHar1.11	3.09	88	Mammalia	319
African Clawed Frog	NCBI	Xenopus_laevis_v10.1	2.74	28.5	Amphibia	352
Coelacanth	NCBI	LatCha_J1.0	2.74	299.48	Sarcopterygii	415
European Perch	NCBI	GENO_Pfluv_1.0	0.95	135	Actinopterygii	429
Smaller Spotted Catshark	NCBI	sScyCan1.2	4.22	63	Chondrichthyes	462
Sea Lamprey	NCBI	kPetMar1.pri	1.09	62.36	Cyclostomata	563
European Lancelet	NCBI	BraLan3	0.47	146	Leptocardii	588
Styela Clava	NCBI	ASM1312258v2	0.34	100	Ascidiacea	588
Bat Star	NCBI	ASM1570657v1	0.61	150	Asteroidea	635
Green Sea Urchin	NCBI	Lvar_3.0	0.87	130	Echinoidea	635
Saccoglossus Kowalevskii	NCBI	Skow_1.1	0.78	7	Enteropneusta	635
Longfin Inshore Squid	NCBI	UCB_Dpea_1	4.6	30	Cephalopoda	708
Sparkling Enope	NCBI	Wsci_v1.0	0.65	24	Cephalopoda	708

Table S9: Enrichment or depletion of UCEs on chromosome short arms. Ns, not significant.

Chromosome	Type	UCEs	Short Arm UCEs	Long Arm UCEs	Expected Short Arm UCEs	Short Arm UCEs / Expected (Fold)	Short Arm Enriched or Depleted
**1**	Metacentric	1306	899	407	638	1.41	Enriched
**2**	Submetacentric	1460	535	922	553	0.97	Ns
**3**	Metacentric	766	365	397	339	1.08	Ns
**4**	Submetacentric	475	102	370	120	0.85	Ns
**5**	Submetacentric	857	65	780	218	0.30	Depleted
**6**	Submetacentric	547	227	317	187	1.21	Enriched
**7**	Submetacentric	604	279	325	220	1.27	Enriched
**8**	Submetacentric	576	148	428	171	0.86	Ns
**9**	Submetacentric	646	228	416	197	1.16	Ns
**10**	Submetacentric	718	97	620	204	0.48	Depleted
**11**	Submetacentric	517	281	236	195	1.44	Enriched
**12**	Submetacentric	513	175	338	128	1.37	Enriched
**13**	Acrocentric	323	0	323	47	0.00	Depleted
**14**	Acrocentric	569	1	568	86	0.01	Depleted
**15**	Acrocentric	498	0	497	85	0.00	Depleted
**16**	Metacentric	433	94	338	169	0.56	Depleted
**17**	Submetacentric	475	54	420	130	0.42	Depleted
**18**	Submetacentric	426	13	411	82	0.16	Depleted
**19**	Metacentric	289	37	252	119	0.31	Depleted
**20**	Metacentric	205	58	142	82	0.71	Depleted
**21**	Acrocentric	115	47	68	27	1.75	Enriched
**22**	Acrocentric	100	3	97	27	0.11	Depleted
**X**	Submetacentric	338	167	170	126	1.33	Enriched
**All**	NA	12756	3875	8842	4107	0.94	Depleted

Supplementary Figures

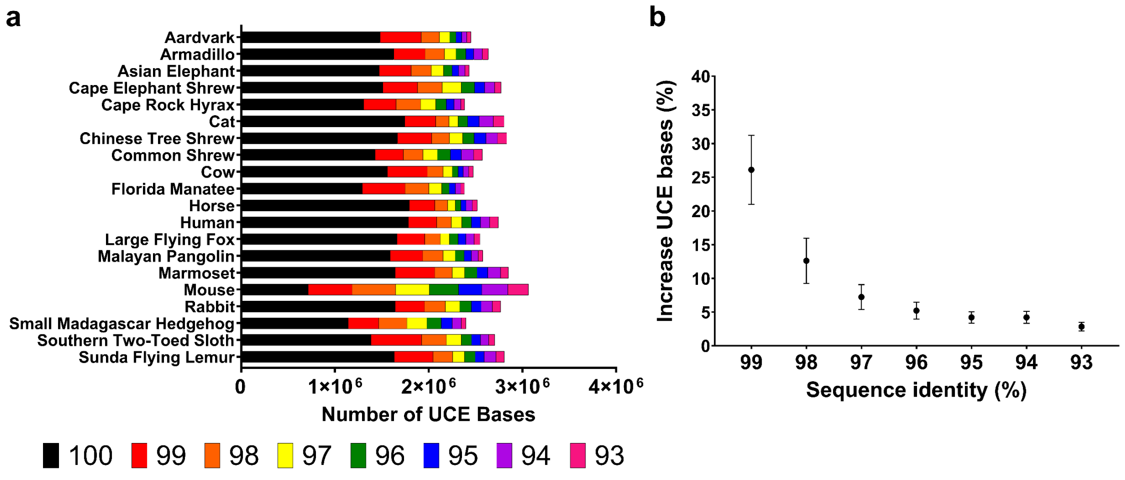

FIG. S1. (**a**) Most UCE bases are found as exact matches in >50% of placental mammal species in the definition set. (**b**) The largest increase in the number of UCE bases is found when allowing for a single mismatch between the UCE sequence and the sequence in any species (mean ± 95% CI).

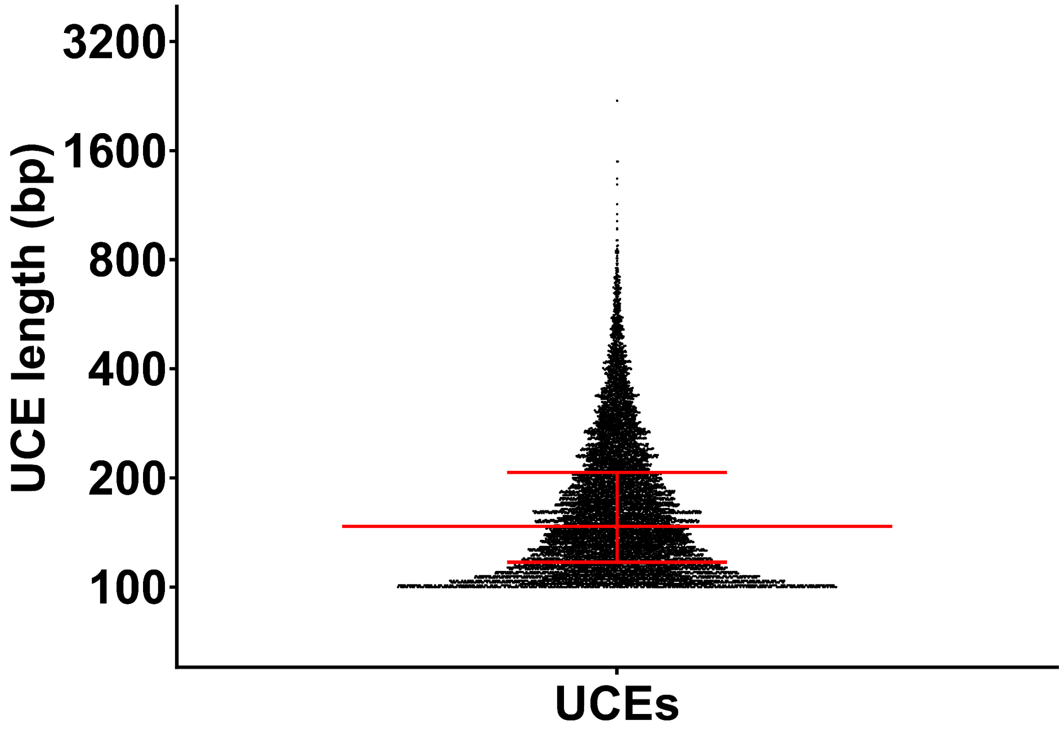

FIG. S2. Scatterplot of UCE base-pair lengths. Red error bars are mean and IQR.

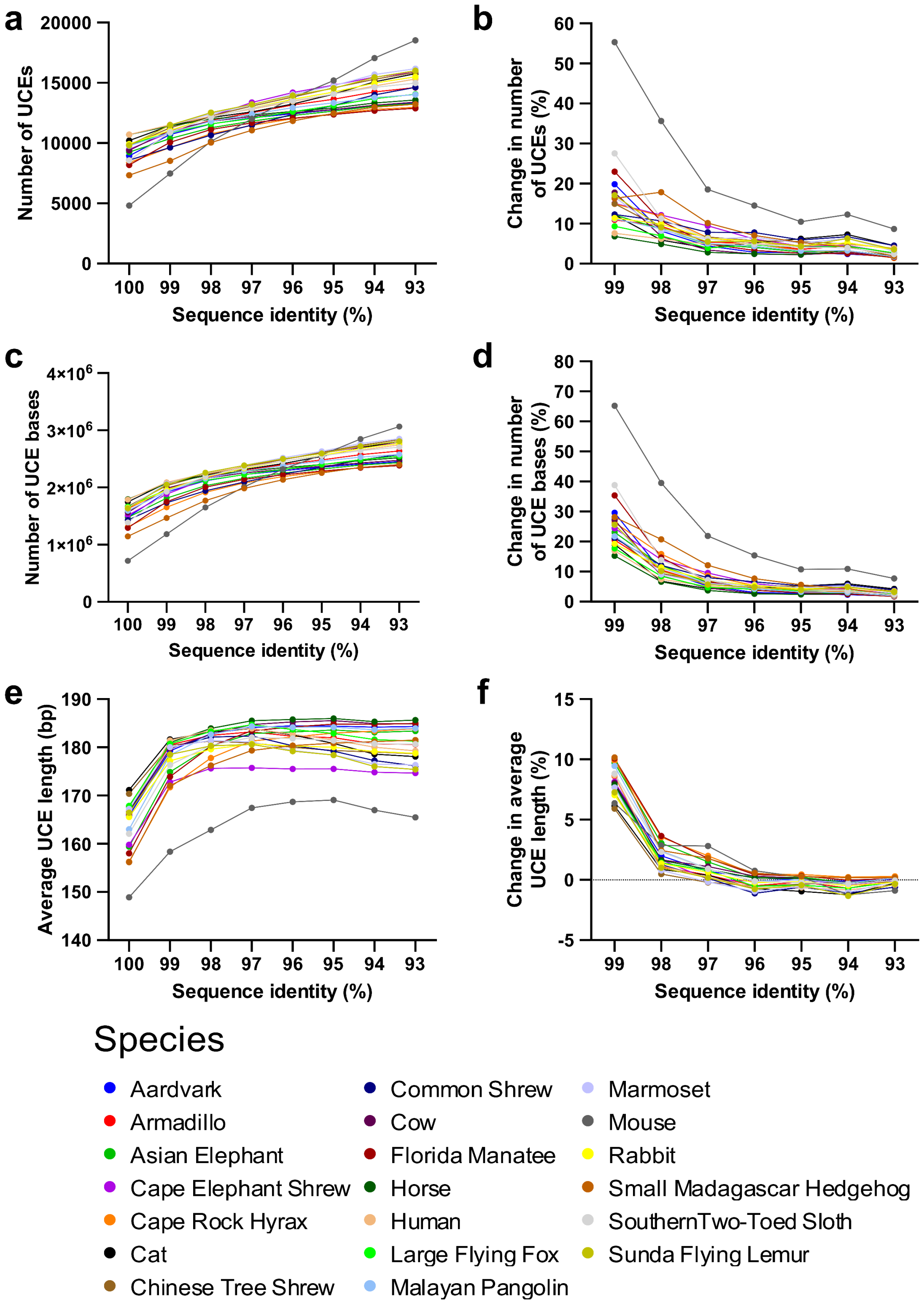

FIG. S3. Placental mammal UCEs in all definition set species. (**a**) The majority of UCEs are found in most species at 100% sequence identity. (**b**) The largest increase in UCEs is observed when sequence identity is decreased to 99%, and plateaus at 96% sequence identity. (**c**) The number of UCE bases and (**d**) the change in UCE bases also follow this pattern. **(e)** The average UCE length increases to reach a maximum in most species at 97% sequence identity, after which (**f**) a slight decrease is observed.

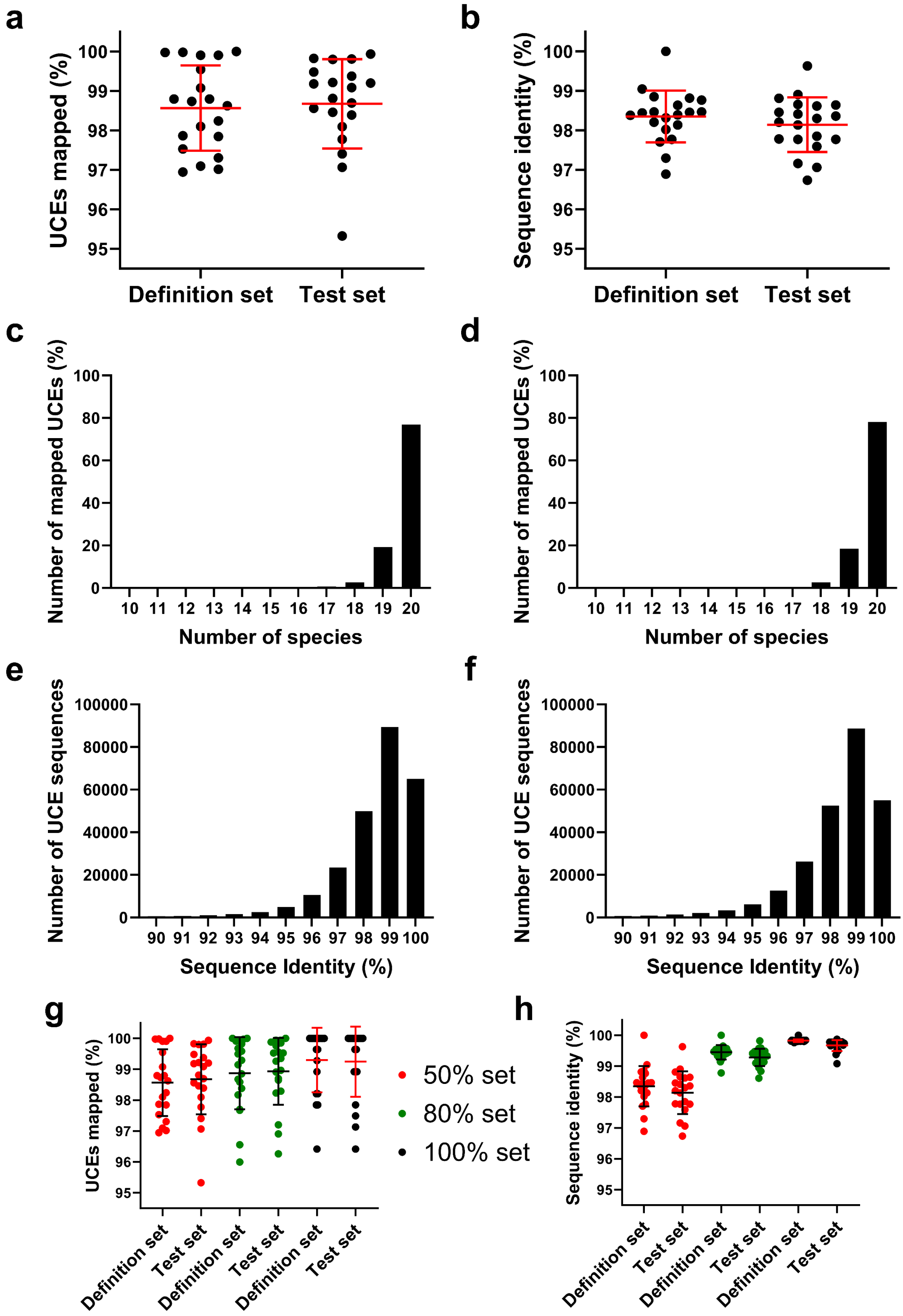

FIG. S4. Validation of UCE sets against a test set of placental mammals. Comparison of (**a**) number of UCEs mapped and (**b**) sequence identity for definition and test sets. UCEs could be mapped in a similar number of species (**c-d**) and with similar sequence identity (**e-f**) in definition (**c,e**) and test (**d,f**) sets. (**g**) Percent of UCEs mapped and (**h**) sequence identity were similar for all 3 UCE sets in both definition and test placentals.

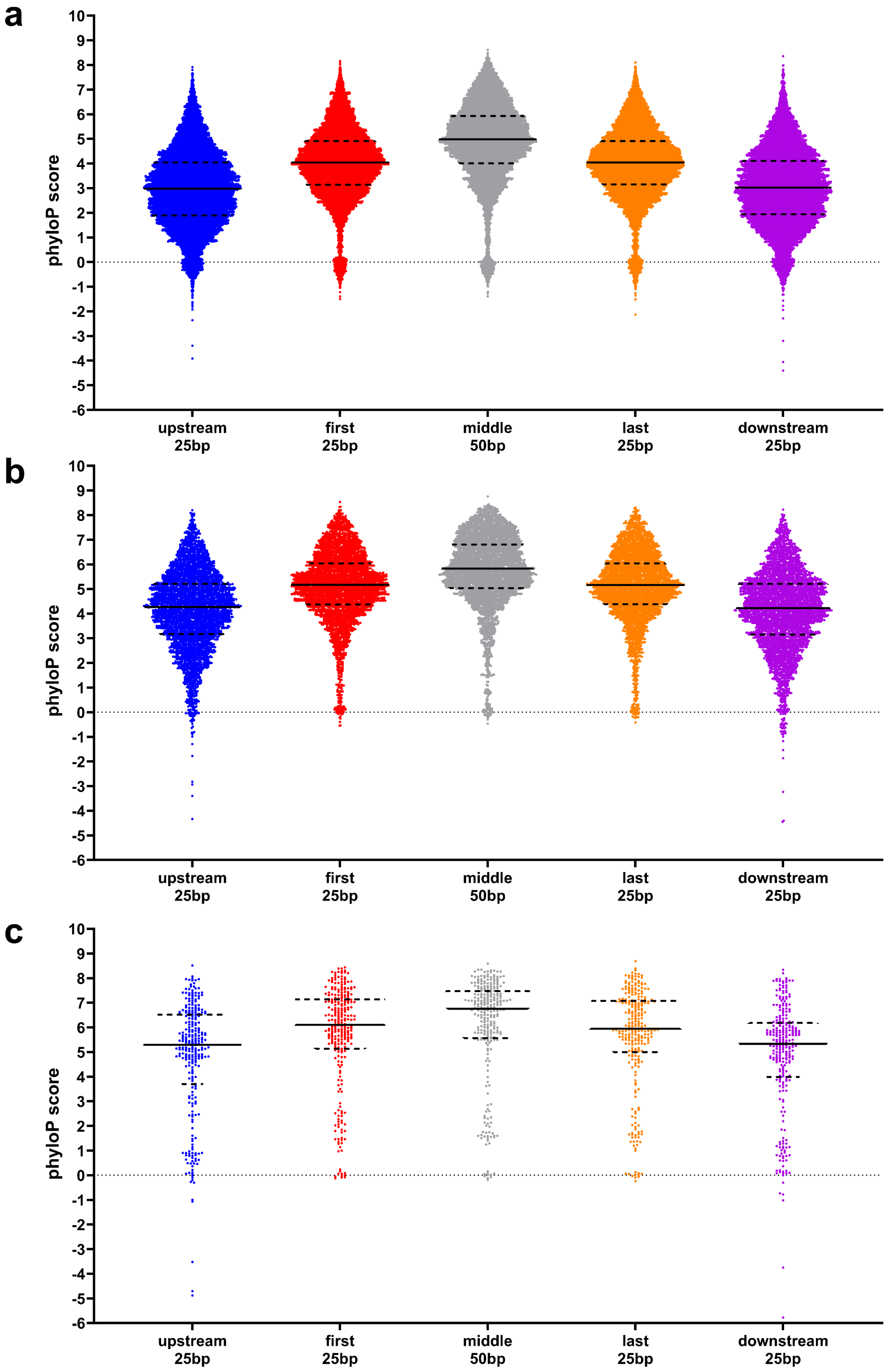

FIG. S5. Conservation of core UCE sequence (middle 50bp) compared to UCE end (first and last 25bp) and flanking sequence (upstream and downstream 25bp) for the (**a**) 50% set (**b**) 80% set (**c**) 100% set.

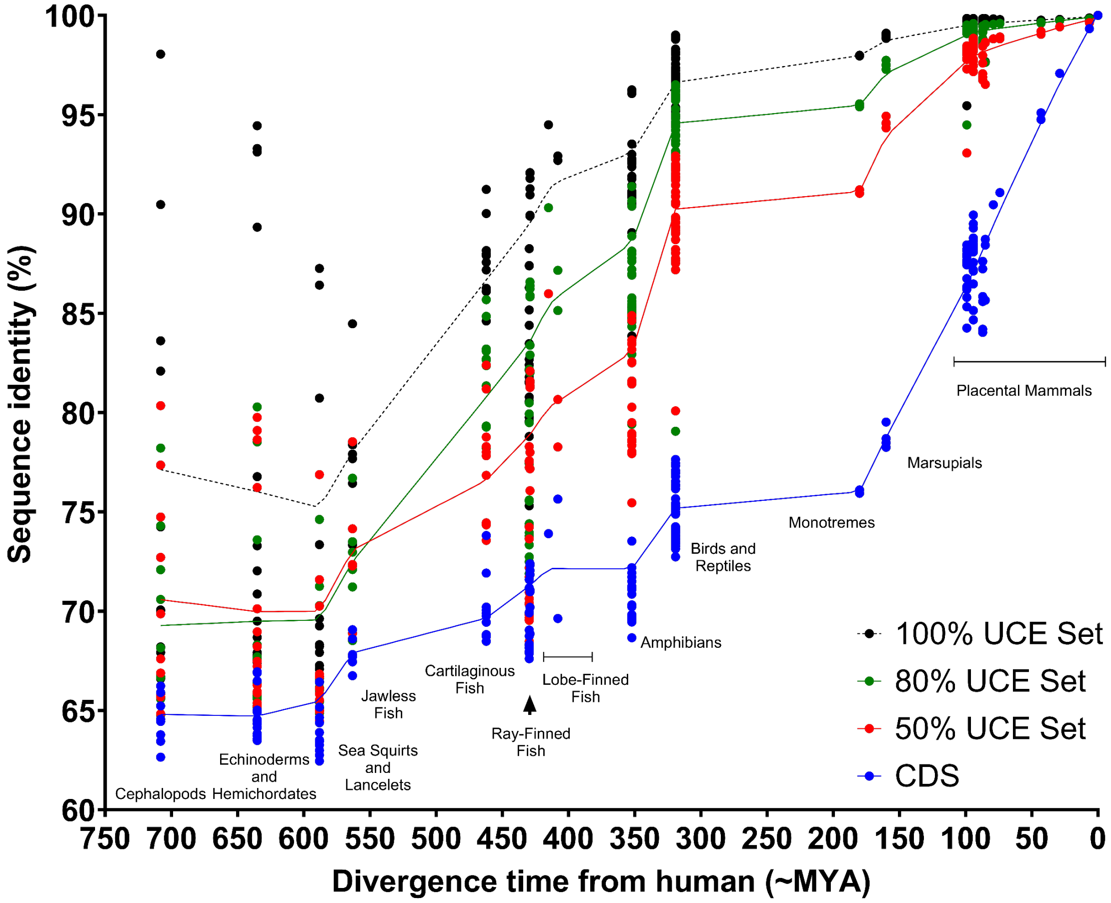

FIG. S6. Sequence identity (%) of UCE sets across 209 species, showing all species. Lines are Lowess medium fitted curves.

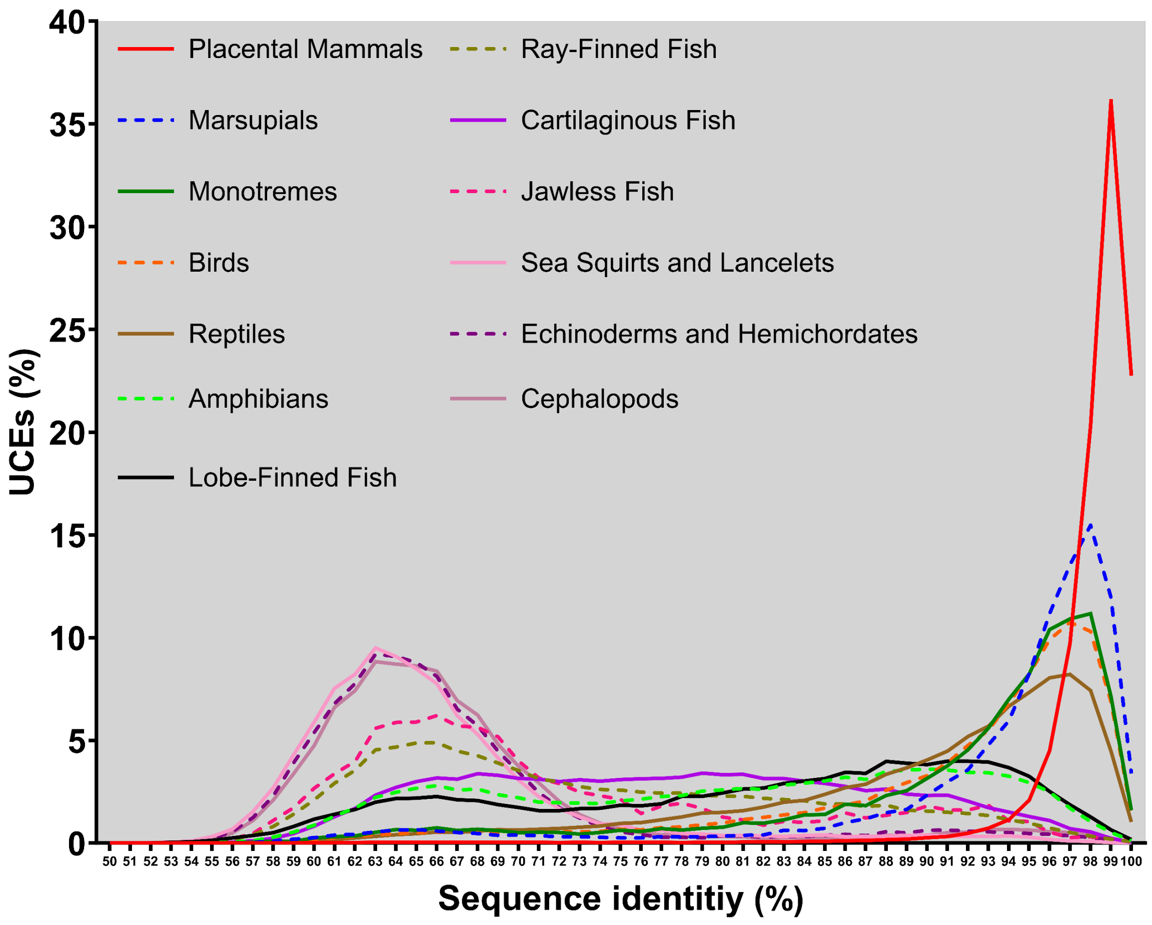

FIG. S7. Bimodal distribution of sequence identity (%) of UCEs (50% set) across clades.

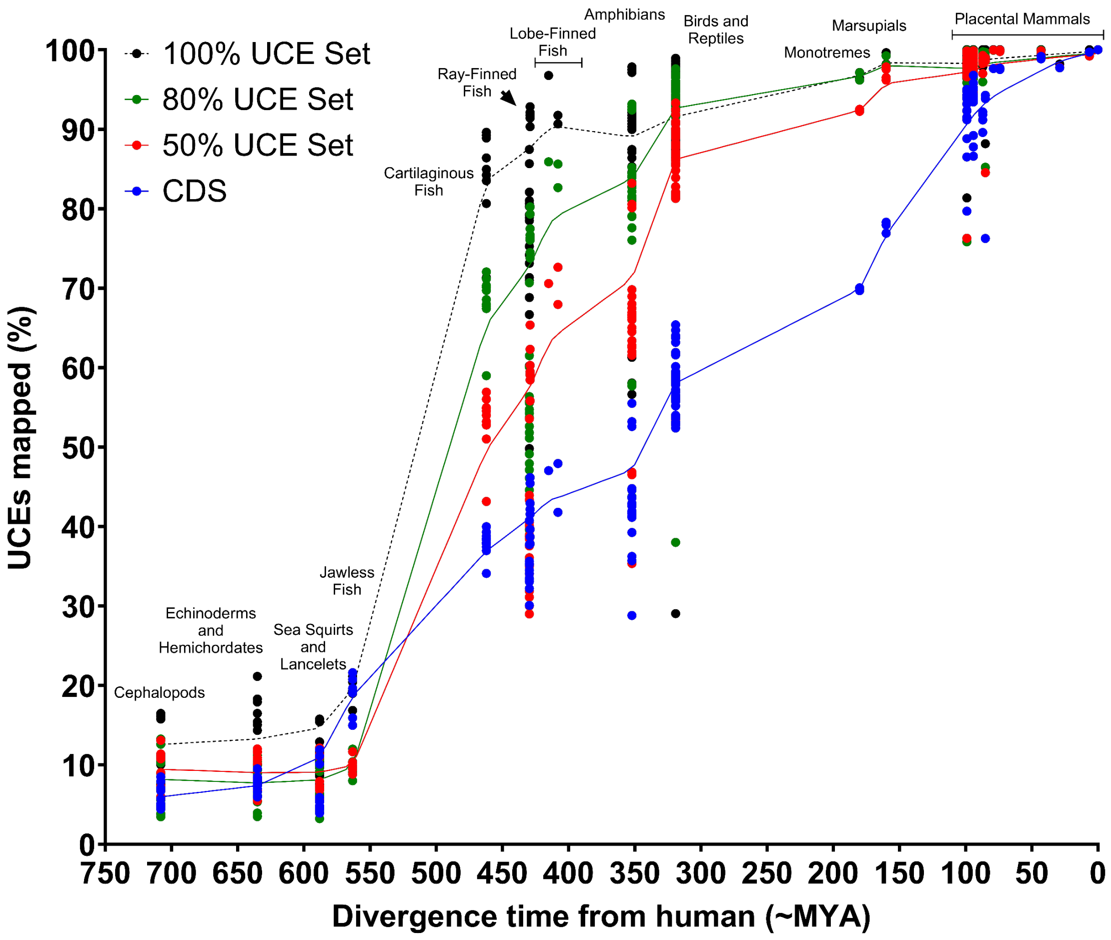

FIG. S8. Number of mapped UCEs (%) of UCE sets across 209 species, showing all species. Lines are Lowess medium fitted curves.

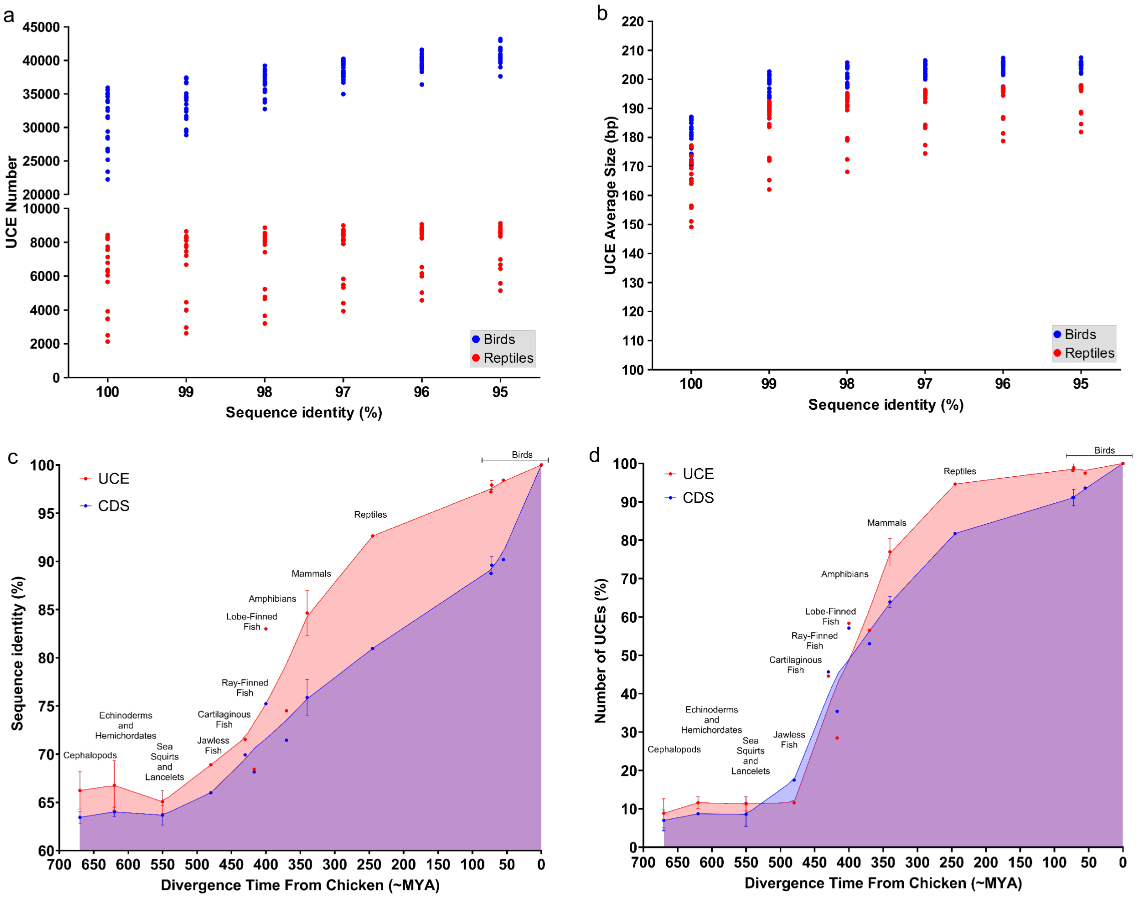

FIG. S9. Bird UCEs. (**a**) Number of UCEs detected in birds and reptiles and (**b**) average size (bp) of UCEs in birds and reptiles. (**c-d**) Chicken sequences of bird UCEs were mapped to a subset of species (see Supplemental Table S8). (**c**) UCE sequence identity (%) and (**d**) mapped UCEs (≥70% sequence identity). The points indicate the mean; vertical bars indicate the standard deviation. Curves are a Lowess medium fitted curve.

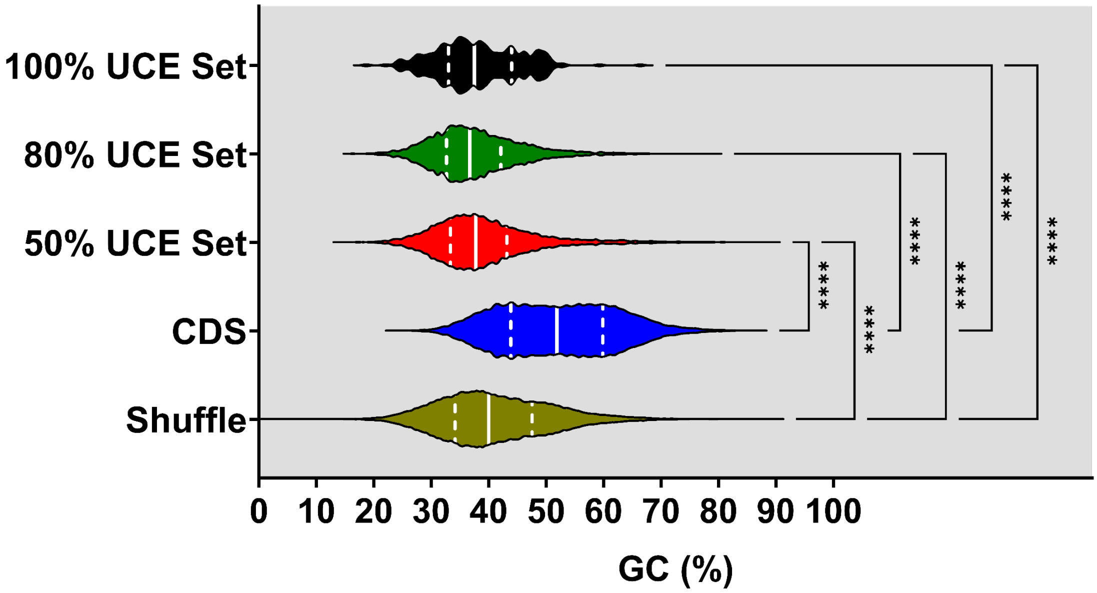

FIG. S10. GC% of UCE sets, protein-coding sequence controls, and shuffled sequence controls. **** p<0.001 Mann Whitney U Test.

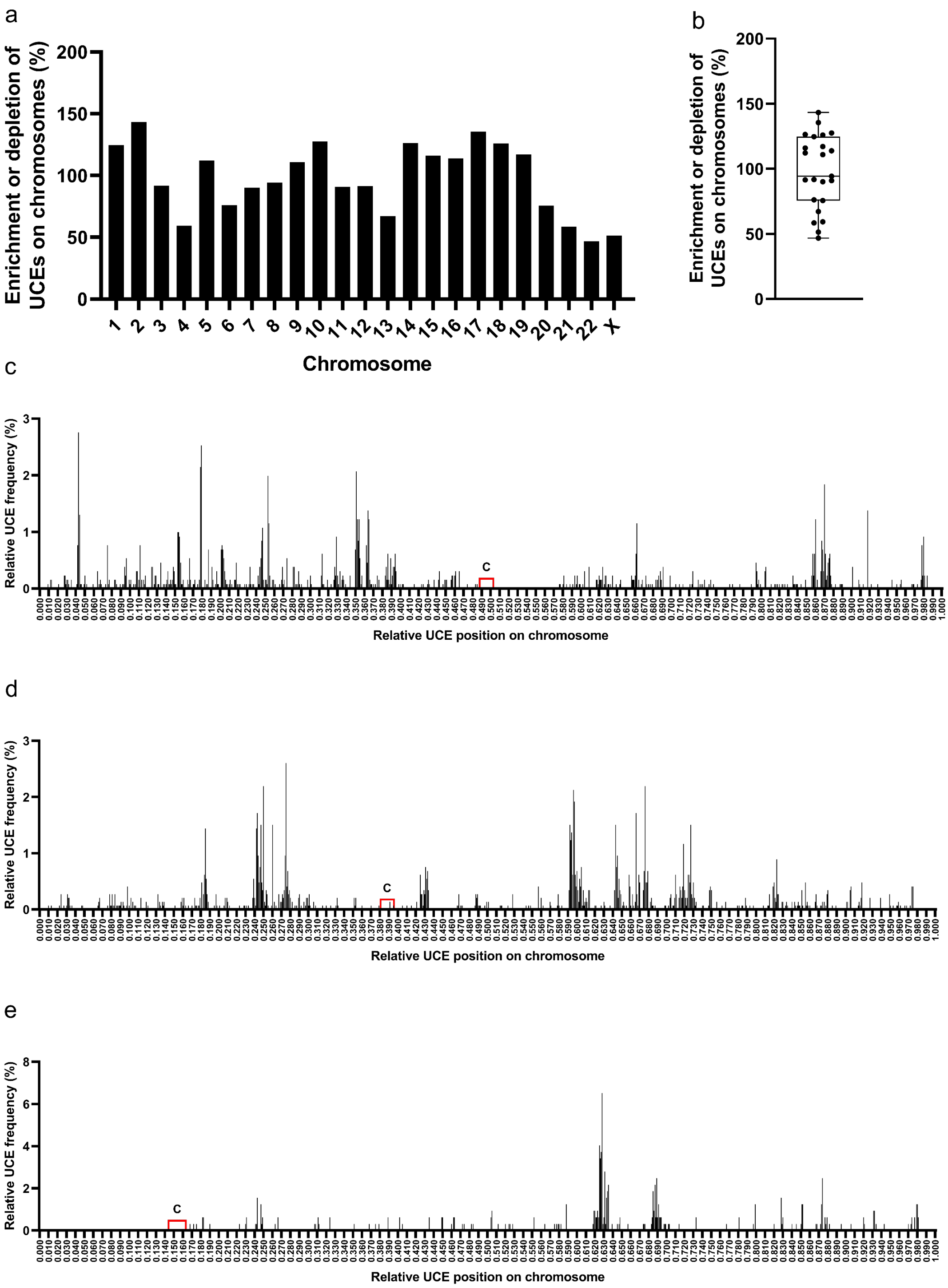

FIG. S11. UCE chromosomal distribution. (**a**) Bar plot of enrichment or depletion of UCEs on human chromosomes. Only 5 of the 23 chromosomes (Y excluded) contained the approximate number expected if UCEs were evenly distributed on each chromosome adjusted for chromosome size (100% ±10%). (**b**) Box and whisker plot of enrichment or depletion of UCEs on chromosomes. Bars plot min and max. Lines plot upper and lower quartiles and median. (**c-e**) Relative UCE position on human chromosomes (**c**) chr1 - metacentric (**d**) chr2 - submetacentric and (**e**) chr13 - acrocentric. UCEs form clusters in all chromosome types. The short arms of acrocentric chromosomes are devoid of UCEs. C (red) is the approximate location of the centromere.

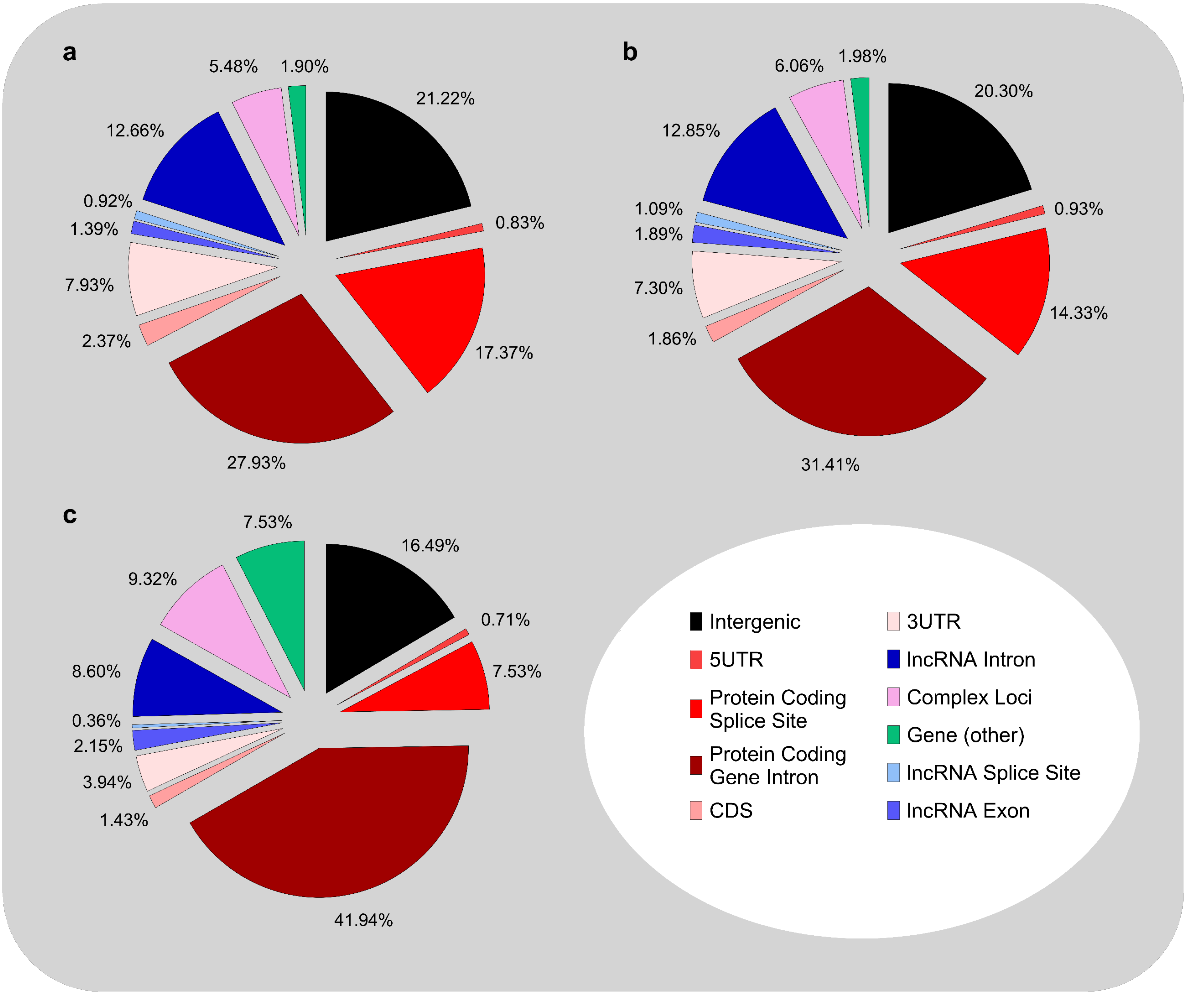

Feature	*Shuffled Average (%)*	*Shuffled SD*	*50% set (%)*	*80% set (%)*	*100% set (%)*	*50% set fold enrichment*	*80% set fold enrichment*	*100% set fold enrichment*
Intergenic	30.52	0.23	21.22	20.30	16.49	0.70	0.67	0.54
5UTR	0.33	0.03	0.84	0.92	0.72	2.55	2.79	2.18
Protein Coding Splice Site	5.72	0.09	17.37	14.33	7.53	3.04	2.51	1.32
Protein Coding Gene Intron	38.85	0.19	27.93	31.41	41.94	0.72	0.81	1.08
CDS	1.16	0.14	2.37	1.86	1.43	2.05	1.61	1.24
3UTR	2.49	0.02	7.93	7.30	3.94	3.19	2.93	1.58
lncRNA Exon	1.37	0.34	1.39	1.89	2.15	1.01	1.38	1.57
lncRNA Splice Site	0.47	0.32	0.92	1.09	0.36	1.96	2.34	0.77
lncRNA Intron	12.03	0.24	12.66	12.85	8.60	1.05	1.07	0.72
Complex Loci	3.99	0.08	5.48	6.06	9.32	1.37	1.52	2.33
Gene (other)	3.07	0.02	1.90	1.98	7.53	0.62	0.64	2.45

FIG. S12. Genomic features containing UCEs for each UCE set. (**a**) 50% UCE set. (**b**) 80% UCE set. (**c**) 100% UCE set. Legend is anti-clockwise around graphs and holds for all figures. UCEs in all sets are predominantly located in introns of protein-coding and lncRNA genes or in intergenic regions. A greater portion of UCEs are located in protein-coding gene introns, other genes, and complex loci in the more stringent sets. Complex loci include gene-intergenic junctions, and loci of overlapping protein-coding and lncRNA genes where the UCE cannot be designated to one gene type (i.e., the UCE is in the intronic region of both gene types, or in exons/splice sites of both gene types). **Table**: Fold enrichment of UCEs in gene features over random shuffled sequences.

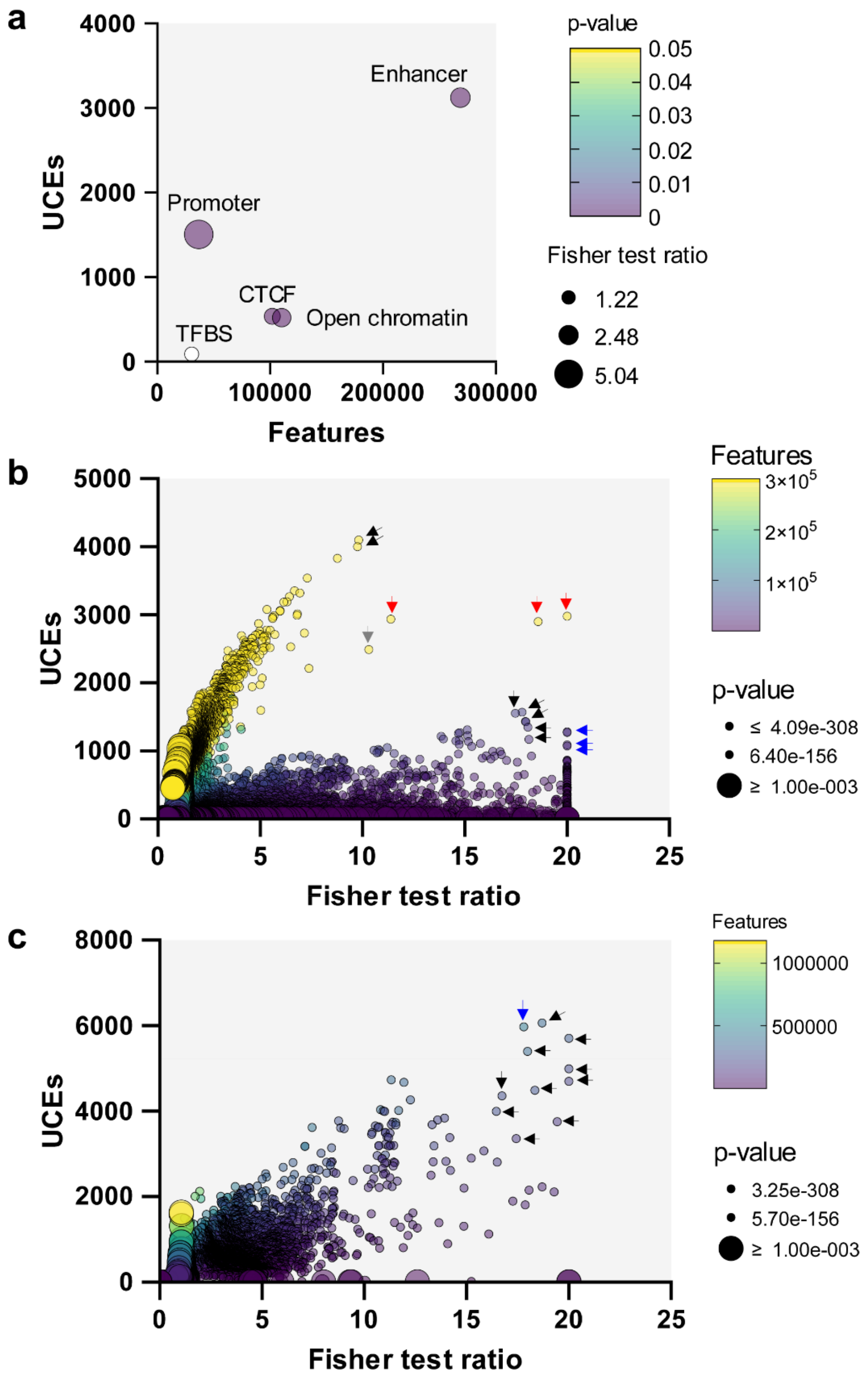

FIG. S13. Enrichment of UCEs in database datasets. (**a**) Enrichment of UCEs in Ensembl regulatory elements. (**b-c**) Enrichment of UCEs in ENCODE (**b**) transcription factor and (**c**) open chromatin datasets. Enrichment was tested using the *bedtools fisher* test. For (**b**) black arrows are neural cells for POLR2AphosphoS5 binding (originated from H1); blue arrows are bipolar neuron for EZH2phosphoT487 binding (originated from GM23338 treated with 0.5 µg/mL doxycycline hyclate for 4 days); red arrows are neural progenitor cell for EZH2 binding (originated from H9); grey arrow is human H1-hESC for SUZ12 binding. For (**c**) black arrows are embryonic brain; blue arrow is embryonic spinal cord.

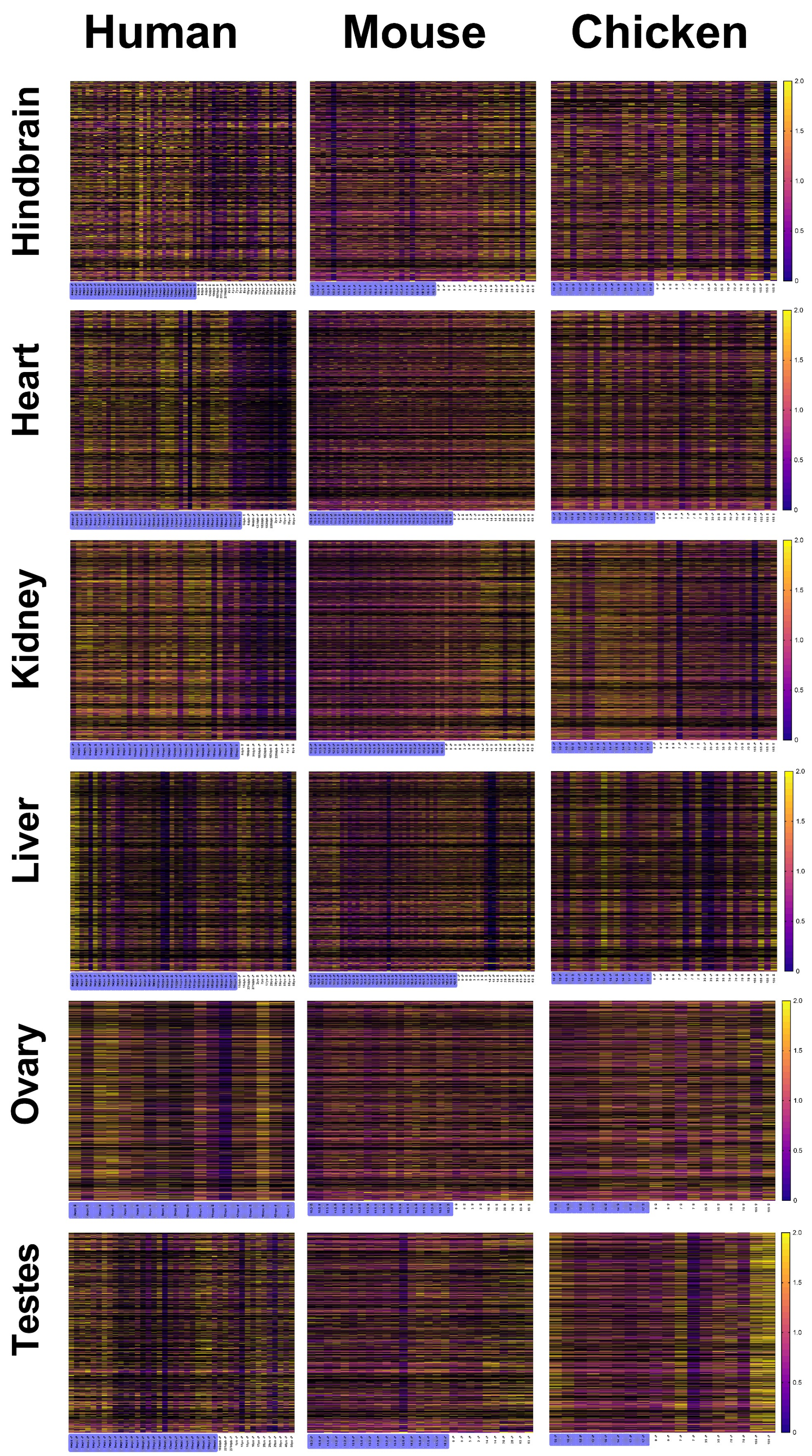

FIG. S14. Expression of UCE loci in human, mouse, and chicken organs during development. Expression values are presented as fold change relative to average sample TPM in the species/organ. Sample names highlighted blue are embryonic timepoints.
