# Supplementary material for: The Evolution of Ultraconserved Elements in Vertebrates
Source: Mol Biol Evol. 2024 Jul 16;41(7):msae146. doi: 10.1093/molbev/msae146 (PMC11276968; doi:10.1093/molbev/msae146)
Supplement: msae146_Supplementary_Data [file msae146_supplementary_data.zip › Supplementary_File_1_Validation_of_dedUCE_and_comparisons_to_other_programs.pdf]

Validation of *dedUCE* and comparisons to other programs

The *dedUCE* program

1. Identifying core k-mers

The algorithm defines a core k-mer as a sequence of at least *k* base pairs in length that are present in ≥X of Y of the input genomes (user defined). This stage uses k-mer hashes to identify core k-mers. First, a k-mer hash is built for each input genome, using the *Jellyfish* hash tool (Marçais & Kingsford, 2011). The hash is then filtered to only non-zero entries and contains a frequency count for all k-mers present in the genome. Next, all entries in the hash are reduced to a count of 1, if present, or 0, if not, giving a binary presence for each k-mer. The hashes are then efficiently summed, again using *Jellyfish*. The resultant hash contains each k-mer present in at least one genome, and the number of unique genomes in which that k-mer occurs. All k-mers with a genome count ≥ X are extracted and marked as core k-mers.

2. Mapping core k-mers

As core k-mers contain only sequence information, not position information, they must then be mapped to the genomes. For this stage, the algorithm defines two parameters which determine mapping behaviour:

- core mismatches: the maximum number of mismatches a core k-mer can have in an aligned position. This setting increases robustness to small changes and allows for inexact UCE matching.
- mapping limit: the maximum number of alignments to which a core k-mer can be associated.

To perform mapping, *dedUCE* allows users to select either *minimap2* (Li, 2018) or *bowtie2* (Langmead & Salzberg, 2012). Alignments are ranked by alignment score and output up to a mapping limit (1000 by default). Where scores are tied at the mapping limit cut-off, the tie is resolved randomly. Mapping limit serves primarily to limit the number of alignments for

repetitive sequences. The default mapping tool is minimap2 for speed purposes but is inexact and may miss some UCEs (although *dedUCE*'s merging algorithm can partially recover from missed mappings). If 100% mapping accuracy of k-mers is required, bowtie2 can be used at the expense of runtime.

3. Merging and UCE identification

The purpose of this step is to identify promising 'runs' of core k-mers and determine if they meet the UCE criteria, which comprises pre-processing core k-mer hits into constant-sized tuples for efficiency, identification of candidate regions in which a UCE may appear, location and extension of valid windows in each candidate region, and identification of valid UCEs.

A candidate region is defined within a single genome and contig, as a run of sorted core k-mer alignments whose start positions are within the maximum gap, G, of the start position of the previous alignment. The maximum gap is derived from the minimum UCE length, L, the minimum UCE sequence identity, I, the length of the core k-mers, k, and a configurable 'buffer' of bases, R (which defaults to 0): $G = L - \lceil IL \rceil + k + R$

The gap allows the region to span over mismatches in potential UCEs. If a UCE is found with the minimum sequence identity possible between two sequences, the worst-case gap is given by the $L - \lceil IL \rceil$ term. The k term accounts for the fact that a single mismatch between two genomes can result in up to k core k-mers vanishing. Since minimap2 is not 100% accurate and may miss short reads, R can somewhat account for accidental dropping of core k-mers. The position information for each genome is read in from the mapping stage, and regions are identified using the *FindCandidateRegions* algorithm. The resulting candidate regions represent parts of the genome which may contain one or more UCEs. After calculating candidate regions for all input genomes, all core k-mers which do not have a hit inside a candidate region are discarded.

Once regions are identified in all genomes, they are checked for the existence of UCEs. A reference genome is chosen from the input genomes (user-specified or selected randomly). When the program parameters require that UCEs appear in all input genomes, only a single reference is required, otherwise multiple references are used (see below). Regions from the reference genome are independently distributed across a pool of processes. A window of

minimum UCE length L bases starts scanning through each region. If the window is a UCE, its length is extended until it is no longer a UCE, otherwise it is moved one base along, and the process repeats.

To determine if a window in the reference genome is a UCE, the reference core k-mer alignments within the window are used as 'keys' to find equivalent runs in the other genomes. For each core k-mer, its positions are found in the other genomes. These positions are then filtered to leave only those which form viable runs, using similar logic to candidate region identification. Finally, for all viable runs, their exact sequences are extracted from the relevant genome and compared to the window's sequence for sequence identity.

If the number of supporting genomes is lower than 100% (i.e., $X < Y$), the window step repeats for a new reference genome, until there is no longer the possibility of UCEs remaining undiscovered. *dedUCE* supports two modes for this step. Both find all UCEs relative to the chosen reference genomes, but their output differs slightly:

i. default: this mode requires a total of $Y - X + 1$ references. When minimum support is less than 100%, the window identification algorithm favours the first non-overlapping UCE found in a valid subset of the genomes. In some cases, this may mean that the UCE output is valid in 3/5 genomes, but if the window had started slightly later it would have found a UCE valid in 4/5 or 5/5 genomes.
ii. exhaustive: this mode requires a total of Y references and runs the window algorithm on every genome as a reference. This identifies overlapping UCEs which appear in different subsets of the input genomes.

4. Extension

Once *dedUCE* has found all UCEs that meet the definition within at least X of Y genomes, it includes an option to find these UCEs in the input genomes with a reduced sequence identity requirement. This is especially useful for finding UCEs in rapidly evolving species. The maximum UCE occurrences parameter controls the mapping limit in this stage. If set to 1, only the positions of unique UCEs will be output. The mapping behaviour with regards to ties is identical to the core k-mer mapping stage. UCE positions are output in BED format, and sequences can be output in FASTA format.

dedUCE validation

To validate *dedUCE*, we sought to replicate lists of UCE from published studies (Table 1) and could identify, on average, ~99% of publish UCEs investigated (Bejerano et al., 2004; Derti, Roth, Church, & Wu, 2006; Makunin, Shloma, Stephen, Pheasant, & Belyakin, 2013; Stephen, Pheasant, Makunin, & Mattick, 2008). Using *dedUCE*, we were able to find all 481 of the originally defined UCEs using the same input genomes and settings as in the original Bejerano *et al.,* (2004) study. We also found all but 1 UCE identified in the expanded sets from Derti *et al.,* (2006). For the larger vertebrate datasets from Makunin *et al.,* (2013), we found 96-100% of published UCEs per list. We also validated *dedUCE* against other vertebrate (Stephen et al., 2008) and invertebrate (Makunin et al., 2013) UCE datasets, and found similarly high congruence. In all bar one dataset, deduce showed increased sensitivity and returned more extra UCEs than were missed. Where possible, we compared UCEs by intersection of coordinates. Where bed files were not available for a dataset, we compared total number of UCEs identified and number of bases (nearest kb).

Table S1: Validation of *dedUCE* using published vertebrate ultraconserved elements (UCEs)

Dataset	***Genomes in dataset***	***Number of UCEs in dataset***	***Number of UCEs identified by dedUCE***	***Percent of UCEs correctly identified by dedUCE***	***UCEs missed by dedUCE***	***Extra UCEs identified by dedUCE***	**UCE bases in dataset (nearest kb)**	**UCE bases dedUCE (nearest kb)**
Bejerano et al., (2004) H-M-R	Human (hg17) Mouse (mm6) Rat (rn3)	481	500	100.0	0	19	126	131
Derti et al., (2006) H-M-D	Human (hg17) Mouse (mm6) Dog (canFam1)	510	515	99.8	1	16	134	138
Derti et al., (2006) H-C	Human (hg17) Chicken (galGal2)	427	436	100.0	0	10	112	116

Derti et al., (2006) Combined dataset	HMR, HMD, and HC datasets	896	922	99.9	1	28	240	249
Makunin et al., (2013) H-D-Mouse	Human (hg18) Dog (canFam2) Mouse (mm8)	5257	5375	99.8	11	140	749	766
Makunin et al., (2013) H-D-Cow	Human (hg18) Dog (canFam2) Cow (bosTau3)	10984	11062	98.9	119	229	1638	1654
Makunin et al., (2013) H-D-Opossum	Human (hg18) Dog (canFam2) Opossum (monDom4)	4031	4124	99.8	8	111	596	609
Makunin et al., (2013) H-D-Platypus	Human (hg18) Dog (canFam2) Platypus (ornAna1)	2677	2539	98.4	42	174	392	374
Makunin et al., (2013) H-D-Chicken	Human (hg18) Dog (canFam2) Chicken (galGal3)	2481	2544	99.6	9	78	368	378
Makunin et al., (2013) H-D-Lizard	Human (hg18) Dog (canFam2) Lizard (anoCar1)	1285	1389	98.1	25	131	185	200

Makunin et al., (2013) H-D-Frog	Human (hg18) Dog (canFam2) Frog (xenTro2)	397	388	96.2	15	8	52	51
Makunin et al., (2013) H-D-Zebrafish	Human (hg18) Dog (canFam2) Zebrafish (danRer4)	22	33	100.0	0	11	3	4
Makunin et al., (2013) H-D-Fugu	Human (hg18) Dog (canFam2) Fugu (fr2)	20	20	100.0	0	0	2	2
Makunin et al., (2013) Combined H-D dataset	Combined dataset of all H-D-Species	12561	12751	99.3	89	319	1934	1966
Stephen et al., (2008) Eutherian dataset	Eutherian set	13736	14766	NA	NA	NA	2131	2258
Stephen et al., (2008) Fish dataset	Fish set	43	45	NA	NA	NA	5	5
Makunin et al., (2013) Mel-Ere-Yak	Drosophila Melanogaster (dm3) Drosophila Erecta (droEre2) Drosophila	19232	20376	99.0	188	1351	2394	2630

	Yakuba (droYak2)							
Makunin et al., (2013) Mel-Ere-Ana	Drosophila Melanogaster (dm3), Drosophila Erecta (droEre2), Drosophila Ananassae (dana_r1.3)	1585	1620	97.5	40	75	186	192
Makunin et al., (2013) Mel-Ere-Pse	Drosophila Melanogaster (dm3), Drosophila Erecta (droEre2), Drosophila Pseudoobscura (dp4)	695	709	98.6	10	24	81	83
Makunin et al., (2013) Mel-Ere-Wil	Drosophila Melanogaster (dm3), Drosophila Erecta (droEre2), Drosophila Willistoni (droWil1)	250	264	99.2	2	16	29	31
Makunin et al., (2013) Mel-Ere-Moj	Drosophila Melanogaster (dm3), Drosophila Erecta (droEre2), Drosophila Mojavensis (droMoj3)	217	236	99.1	2	21	26	30

Makunin et al., (2013) Mel-Ere-Vir	Drosophila Melanogaster (dm3), Drosophila Erecta (droEre2), Drosophila Virilis (droVir3)	221	232	98.2	4	15	26	27
Makunin et al., (2013) Mel-Ere-Gri	Drosophila Melanogaster (dm3), Drosophila Erecta (droEre2), Drosophila Grimshawi (droGri2)	213	226	99.5	1	14	25	27
Makunin et al., (2013) Combined Drosophila dataset	Combined dataset of all Mel-Ere-Species	19438	20579	99.0	193	1352	2421	2590
Makunin et al., (2013) Combined Sophophora dataset	Combined dataset of 4 comparisons<br>Mel-Ere-Ana<br>Mel-Ere-Pse<br>Mel-Yak-Ana<br>Mel-Yak-Pse	2126	2212	97.7	48	134	249	263

Comparisons to other tools

There have been many different approaches developed to identify UCEs, however, *dedUCE* offers significant improvements over other programs. Below we provide a brief explanation of the advantages *dedUCE* offers compared to other common tools for identifying UCEs. We also used the Makunin et al., (2013) vertebrate datasets to compare *dedUCE* to other methods used for identification of UCEs (Armstrong et al., 2020; Christmas et al., 2023; Faircloth, 2016) (Table 2). In brief, we found that *PHYLUCE* identified ~86-95% of dataset UCE loci when simulating reads at 50X coverage and took ~4x computing time of *dedUCE*. We found that *Progressive Cactus* contained multi-alignments at ~65-99.4% of dataset UCE loci, with low percentages in fish alignments, and took ~1120x computing time of *dedUCE*.

PHYLUCE

Arguably the most popular tool for identifying UCE loci, *PHYLUCE* (Faircloth, 2016) is primarily designed for inferring phylogeny from target-enriched loci DNA sequencing, but also offers a method for harvesting UCE loci from reference genomes. This method involves generating simulated reads (without errors) from the genomes of interest and aligning those simulated reads to a reference species genome. The main disadvantage of this program is the requirement of a reference genome, which limits discovery of UCE loci to those present in the reference. In contrast *dedUCE* does not require the use of a reference genome, instead identifying all UCEs that occur at or above a given proportion of genomes, decreasing the effect a single species may have on the analysis. There are also other minor disadvantages to the program. Firstly, the program requires significant filtering of the loci to exclude those in repeat elements, or the use of masked genomes to avoid this issue. By using reads simulated, you generate many reads from repeat elements which increases mapping time unnecessarily. If soft-masked genomes are used, loci then need to be filtered to exclude loci with >X% masked bases (suggested 25% by *PHYLUCE*). While the use of hard-masked genomes avoids this problem, it also limits discovery of UCEs to regions containing no repeat elements. *dedUCE* tackles this problem by allowing the user to set filters for number of times core k-mers and UCEs can map to a genome. This allows the user to discover UCEs that partially overlap with repeat sequence without significantly increasing mapping time, and also avoids the need to post-filter loci. The use of simulated reads presents another problem, that being the level of coverage you need to simulate. Whilst the

PHYLUCE tutorial suggests simulating reads at 2X coverage (to replicated low coverage DNA sequencing), we found that 50X coverage was required to approach the performance of *dedUCE* against published datasets (Table 2). This resulted in a significant increase in runtime. Finally, we found that the program was not user friendly given the requirements of specific directory structures and significant hands-on time. In contrast, *dedUCE* only requires all genome fasta to be in a single directory, everything else is automated for ease of use.

Multi-alignment approach – Progressive Cactus

As an exemplar for multiple-genome alignment approaches to UCE discovery, we compared dedUCE to *Progressive Cactus*, a multiple-genome aligner that allows for reference free alignment of thousands of genomes (Armstrong et al., 2020). Whilst multi-alignment approaches offer significant advantages, such as the ability to filter (or not) for syntenic blocks, the main disadvantage is runtime and resource usage, which *dedUCE* improves significantly. We found that *dedUCE* outperformed *Progressive Cactus* against previous UCE datasets and was significantly faster than the multi-alignment (Table 2).

Table S2: Comparison of *dedUCE* to *PHYLUCE* and *Progressive Cactus*.

Dataset	***Dataset UCEs***	***Program***	***UCEs identified***	***UCEs % identified***	***UCEs missed***	***CPU time (h:m:s)***	***Memory used (kb)***
Makunin et al., (2013) H-D-Mouse	5257	dedUCE	5246	99.8	11	02:51:35	125831048
		PHYLUCE	4817	91.6	440	10:05:54	125831168
		Progressive Cactus	5205	99	52	2479:23:27	197461684
Makunin et al., (2013) H-D-Cow	10984	dedUCE	10865	98.9	119	02:50:53	125831168
		PHYLUCE	9998	91.0	986	08:55:24	125829120
		Progressive Cactus	10836	98.7	148	2648:24:28	275290676
	4031	dedUCE	4023	99.8	8	02:23:14	125829120

Makunin et al., (2013) H-D-Opossum		PHYLUCE	3763	93.4	268	11:00:36	125829120
		Progressive Cactus	3976	98.6	55	3685:10:43	267010672
Makunin et al., (2013) H-D-Platypus	2677	dedUCE	2635	98.4	42	01:48:57	125829120
		PHYLUCE	2492	93.1	185	08:03:19	125831168
		Progressive Cactus	2610	97.5	67	2438:52:35	213494276
Makunin et al., (2013) H-D-Chicken	2481	dedUCE	2472	99.6	9	01:40:10	122482020
		PHYLUCE	2359	95.1	122	07:02:30	125831168
		Progressive Cactus	2384	96.1	97	2473:31:26	222457824
Makunin et al., (2013) H-D-Lizard	1285	dedUCE	1260	98.1	25	01:42:17	125829120
		PHYLUCE	1211	94.2	74	07:45:34	125829120
		Progressive Cactus	1241	96.6	44	1971:18:16	142640160
Makunin et al., (2013) H-D-Frog	397	dedUCE	382	96.2	15	02:01:27	125833172
		PHYLUCE	372	93.7	25	07:27:23	125829120
		Progressive Cactus	361	90.9	36	2040:32:56	153297588
Makunin et al., (2013) H-D-Zebrafish	22	dedUCE	22	100.0	0	02:05:19	125831172
		PHYLUCE	19	86.4	3	07:55:25	125831168
		Progressive Cactus	16	72.7	6	2000:14:01	137551440
	20	dedUCE	20	100.0	0	01:46:23	125829136

Makunin et al., (2013) H-D-Fugu		PHYLUCE	18	90.0	2	06:15:46	125831168
		Progressive Cactus	13	65.0	7	1361:55:08	138881928
Makunin et al., (2013) Combined H-D dataset	12561	dedUCE	12472	99.3	89	N/A	N/A
		PHYLUCE	11490	91.5	1071	N/A	N/A
		Progressive Cactus	12485	99.4	76	N/A	N/A

References

Armstrong, J., Hickey, G., Diekhans, M., Fiddes, I. T., Novak, A. M., Deran, A., . . . Paten, B. (2020). Progressive Cactus is a multiple-genome aligner for the thousand-genome era. *Nature, 587*(7833), 246-251. doi:10.1038/s41586-020-2871-y

Bejerano, G., Pheasant, M., Makunin, I., Stephen, S., Kent, W. J., Mattick, J. S., & Haussler, D. (2004). Ultraconserved elements in the human genome. *Science, 304*(5675), 1321-1325. doi:10.1126/science.1098119

Derti, A., Roth, F. P., Church, G. M., & Wu, C. T. (2006). Mammalian ultraconserved elements are strongly depleted among segmental duplications and copy number variants. *Nat Genet, 38*(10), 1216-1220. doi:10.1038/ng1888

Faircloth, B. C. (2016). PHYLUCE is a software package for the analysis of conserved genomic loci. *Bioinformatics, 32*(5), 786-788. doi:10.1093/bioinformatics/btv646

Langmead, B., & Salzberg, S. L. (2012). Fast gapped-read alignment with Bowtie 2. *Nat Methods, 9*(4), 357-359. doi:10.1038/nmeth.1923

Li, H. (2018). Minimap2: pairwise alignment for nucleotide sequences. *Bioinformatics, 34*(18), 3094-3100. doi:10.1093/bioinformatics/bty191

Makunin, I. V., Shloma, V. V., Stephen, S. J., Pheasant, M., & Belyakin, S. N. (2013). Comparison of ultra-conserved elements in drosophilids and vertebrates. *PLoS One, 8*(12), e82362. doi:10.1371/journal.pone.0082362

Marçais, G., & Kingsford, C. (2011). A fast, lock-free approach for efficient parallel counting of occurrences of k-mers. *Bioinformatics, 27*(6), 764-770. doi:10.1093/bioinformatics/btr011

Stephen, S., Pheasant, M., Makunin, I. V., & Mattick, J. S. (2008). Large-scale appearance of ultraconserved elements in tetrapod genomes and slowdown of the molecular clock. *Mol Biol Evol, 25*(2), 402-408. doi:10.1093/molbev/msm268
